# Single-transcript multiplex *in situ *hybridisation reveals unique patterns of dystrophin isoform expression in the developing mammalian embryo

**DOI:** 10.12688/wellcomeopenres.15762.2

**Published:** 2020-07-20

**Authors:** John C. W. Hildyard, Abbe H. Crawford, Faye Rawson, Dominique O. Riddell, Rachel C. M. Harron, Richard J. Piercy

**Affiliations:** 1 Department of Clinical Science and Services, Royal Veterinary College, London, Camden, London, NW1 0TU, UK

**Keywords:** Dystrophin, RNAscope, In-situ hybridisation, Embryogenesis, Development, Expression, Dp427, Dp140, Dp71, DMD, DeltaE50-MD, Muscle, Nerve, Brain

## Abstract

**Background:** The dystrophin gene has multiple isoforms: full-length dystrophin (dp427) is principally known for its expression in skeletal and cardiac muscle, but is also expressed in the brain, and several internal promoters give rise to shorter, N-terminally truncated isoforms with wider tissue expression patterns (dp260 in the retina, dp140 in the brain and dp71 in many tissues). These isoforms are believed to play unique cellular roles both during embryogenesis and in adulthood, but their shared sequence identity at both mRNA and protein levels makes study of distinct isoforms challenging by conventional methods.

**Methods:** RNAscope is a novel *in-situ* hybridisation technique that offers single-transcript resolution and the ability to multiplex, with different target sequences assigned to distinct fluorophores. Using probes designed to different regions of the dystrophin transcript (targeting 5', central and 3' sequences of the long dp427 mRNA), we can simultaneously detect and distinguish multiple dystrophin mRNA isoforms at sub-cellular histological levels. We have used these probes in healthy and dystrophic canine embryos to gain unique insights into isoform expression and distribution in the developing mammal.

**Results:** Dp427 is found in developing muscle as expected, apparently enriched at nascent myotendinous junctions. Endothelial and epithelial surfaces express dp71 only. Within the brain and spinal cord, all three isoforms are expressed in spatially distinct regions: dp71 predominates within proliferating germinal layer cells, dp140 within maturing, migrating cells and dp427 appears within more established cell populations. Dystrophin is also found within developing bones and teeth, something previously unreported, and our data suggests orchestrated involvement of multiple isoforms in formation of these tissues.

**Conclusions:** Overall, shorter isoforms appear associated with proliferation and migration, and longer isoforms with terminal lineage commitment: we discuss the distinct structural contributions and transcriptional demands suggested by these findings.

## Introduction

**Figure 1.**
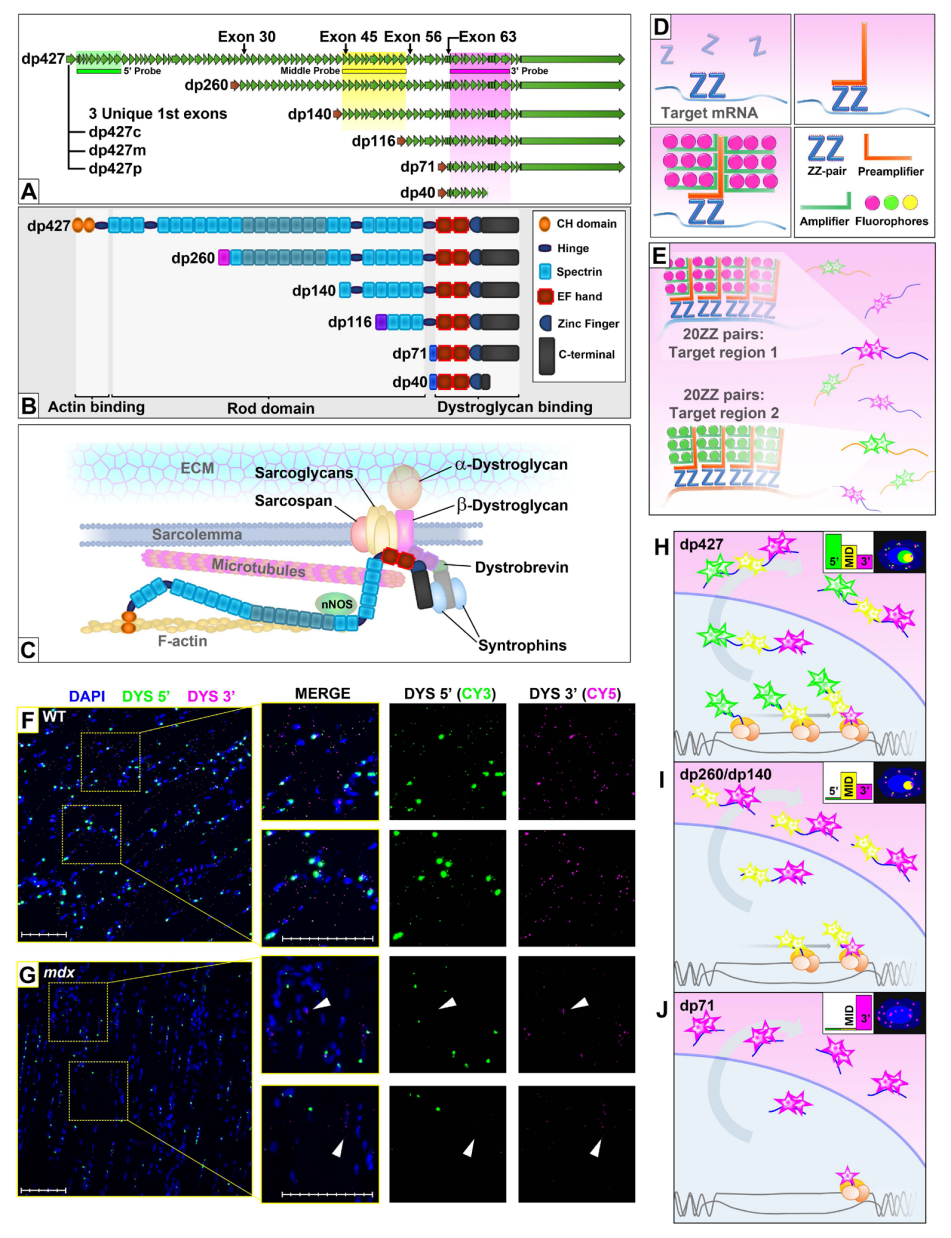
Dystrophin isoforms and RNAscope multiplex ISH. ( **A**) Dystrophin transcripts: the dystrophin gene has multiple promoters, three generating full-length isoforms (dp427c,m,p) and four internal promoters giving rise to shorter isoforms (dp260, dp140, dp116 and dp71). A further short isoform (dp40) is produced from alternate splicing of dp71. Target binding sites of the multiplex probes (5’, middle, 3’) used in this study are indicated. ( **B**) Dystrophin proteins: dp427 carries multiple functional domains, subsets of which are retained by the shorter isoforms. The darker shading of spectrin repeats 11–17 (present in dp427 and dp260) indicates the additional actin-binding motif of these isoforms. ( **C**) Dp427 is found beneath the muscle sarcolemma as part of the multimeric dystrophin-associated glycoprotein complex (DAGC), forming a physical link between the f-actin cytoskeleton and the muscle extracellular matrix. ( **D**, **E**) The RNAscope ISH method uses ‘ZZ pair’ probes with an enzyme-linked amplifier-tree strategy to localise and immobilise high concentrations of fluorophore in a probe-specific manner, allowing single transcript resolution and labelling of multiple target transcripts/sequences. ( **F**, **G**) Dystrophin 5’/3’-targeted multiplex ISH in mouse muscle: in healthy muscle ( **F**) nascent dp427 mRNAs within nuclei label strongly with 5’ probe alone, while mature cytoplasmic dp427 transcripts are labelled with smaller punctate foci of both 5’ and 3’ probes in close apposition. In dystrophic *mdx* muscle ( **G**) cytoplasmic 5’ labelling is absent and nuclear 5’ signal is reduced, though nuclei associated with 3’ foci only (dp71) are infrequently observed (arrowheads). ( **H**, **I**, **J**) Expected behaviour of dystrophin isoforms with triplex ISH probes: high numbers of nascent transcripts within cells expressing dp427 ( **H**) will produce strong 5’ nuclear labelling (green), modest middle probe nuclear labelling (yellow) and infrequent nuclear 3’ labelling (magenta), with small cytoplasmic foci of all three probes. Cells expressing dp140 ( **I**) will exhibit a similar pattern for middle and 3’ probes, but 5’ signal will be absent. Cells expressing dp71 ( **J**) will show small foci of 3’ probe only in all cellular compartments. Full-size figure can be found in the *Underlying data* ^35^.

Dystrophin is one of the largest genes in the genome: at approximately 2.3 million base-pairs (Mbp) in length, and comprising 79 exons, this single locus occupies fully 1.5% of the X chromosome where it resides ^1^. The gene is also ancient: as part of the dystrophin/dystrobrevin/dystrotelin superfamily, it likely predates metazoan phylogenesis ^2^, and at least one dystrophin-like gene is found in all metazoa. Transcription of this lengthy gene requires ~16 hours, with co-transcriptional splicing ^3^, ultimately yielding a transcript of 14 kb. In skeletal muscle this mature mRNA is translated to produce a 427 KDa dystrophin protein (dp427) ^4^, which plays a key role in maintaining the integrity of the muscle sarcolemma: absence of this protein results in muscle vulnerable to contraction-induced injury, leading to the fatal X-linked muscle-wasting disease, Duchenne muscular dystrophy (DMD). Dystrophin plays many additional roles across different tissues, however, with unconventional regulation of expression ( Figure 1A): dp427 can be transcribed from three discrete promoters with distinct expression patterns, yielding muscle (dp427m), cortical (dp427c) and Purkinje (dp427p) isoforms expressed in muscle, brain and cerebellar Purkinje cells, respectively ^5– 8^. A further four internal promoters lead to shorter isoforms, with similarly distinct expression: dp260 in the retina ^9^, dp140 in the brain (and also the eye) ^10^, dp116 in Schwann cells ^11^, and finally dp71, expressed near-ubiquitously (but notably absent from skeletal muscle ^12^). Such specific expression profiles imply distinct functional contributions, though further subtleties complicate interpretation: dp427m has been reported in glial cells ^13^, dp427c in the eye, and dp427p in skeletal muscle ^14^, and moreover many (potentially all) dystrophin isoforms might be alternatively spliced at the extreme 3’ end (typically via omission of exons 71–74 and also 78) ^15, 16^. Inclusion of intron 70 during transcription of dp71 introduces a novel stop codon and polyA site leading to an even shorter isoform, dp40, expressed with a similar profile to dp71 (but at lower levels) ^16^.

### Dystrophin isoforms in development

Dystrophin is expressed during embryonic/foetal growth, and dystrophin isoforms alter in their expression throughout development, suggesting they play key roles in this process. Dp140 is found in the adult brain (primarily the cerebellum), but levels of this isoform are markedly higher and more widespread in embryonic neural tissues ^17^, and dp140 is also found in the S- and comma-shaped bodies of the developing (but not adult) kidney ^18^. Dp71 is particularly enriched in developing epithelial tissues, and within the lung and embryonic eye ^19, 20^. Moreover, while expression of this short isoform is essentially absent in mature myofibres, dp71 is found within proliferating myoblasts, only replaced by dp427 during differentiation ^21^. Expression of each dystrophin isoform is clearly tightly orchestrated, yet the specific roles of each isoform at different developmental stages and in adulthood remain at present poorly understood.

### The dystrophin protein: structure and function

The dystrophin protein is functionally complex ( Figure 1B, C): full length dp427 has three principal domains ^22^: an actin-binding N-terminus, a long multifunctional rod domain of 24 spectrin-like repeats, and a cysteine-rich C terminus that interacts with β-dystroglycan and other transmembrane/membrane associated proteins such as dystrobrevin, syntrophin and the sarcoglycans, forming the dystrophin-associated glycoprotein complex (DAGC) ^23, 24^. The N and C termini are critical for dp427 function: loss of either results in DMD, while mutations causing internal truncations that retain N- and C-terminal domains typically result in the milder Becker muscular dystrophy (BMD). Recombinant ‘microdystrophin’ constructs lacking the majority of the rod domain ameliorate disease in animal models ^25– 28^, indicating that a physical link between cytoskeletal actin and the extracellular matrix (ECM) is essential for muscle stability. All of the shorter isoforms carry the C-terminal domain (partially in the case of dp40) while none retains the actin-binding N-terminus; each also carries a unique subset of the 24 spectrin-like repeats of the dp427 rod domain, and hence, presumably, some rod domain functions. Repeats 11–17 form an additional actin-binding domain ^29, 30^, and repeats 16–17 further bind neuronal nitric oxide synthase (nNOS) ^31^, allowing muscle contraction to elicit local vasodilation. Of the shorter isoforms, only dp260 retains both these capabilities (and is able to form a weaker physical link: transgenic expression of dp260 in dystrophic muscle can partially compensate for absence of dp427 ^32^). Repeats 20–23 bind and organise microtubule networks ^33, 34^, a property presumably retained by dp260, dp140 and potentially even dp116, but not by dp71/dp40.

### Study of dystrophin isoform expression

Deciphering the unique developmental contributions of each isoform is non-trivial: all isoform transcripts carry unique first exons (dp40 aside), but otherwise share canonical dystrophin sequence (indeed dp427m, c and p differ by only 11, 3 and 7 unique amino acids, respectively), leading to very high identity at both nucleotide and protein level. PCR targeted to unique first exon sequence can distinguish isoforms at mRNA level, and western blotting with antibodies raised to the C terminus can identify isoform proteins by virtue of size, but these approaches typically use tissue homogenates, necessarily losing spatial information. Transcriptomic approaches (such as microarray analysis) are seldom designed with such isoform-level analysis in mind, generally either capturing 5’ sequence (thus dp427 only), or universally shared 3’ sequence. Comprehensive sequence coverage offered by RNAseq permits more nuanced analysis, and functional roles can be inferred by comparison with expression of genes of known function: elegant work by Doorenweerd *et al.* ^17^ showed that dp140 expression in the developing brain aligns well with that of genes involved in axonal migration. Again however, tissue homogenates are the norm for such techniques, and even precisely selected regions of tissue might contain multiple cell types that will necessarily be assessed collectively.

Histological studies, in contrast, offer high-resolution spatial detail, but face different challenges: the near-complete sequence-identity means antibodies specific for short isoforms are difficult to raise (as noted, C-terminal antibodies recognise all isoforms), and moreover many tissues express very low levels of protein, close to or below the limit of detection. Transgenic C-terminal fusion of eGFP reporters ^36^ permits study of expression even *in-vivo*, but again without isoform specificity. Similarly, transgenic insertion of beta-galactosidase cassettes after the unique first exon can provide valuable high-sensitivity expression data even in a whole-mount context (as shown with dp71 by Sarig *et al.* ^19^), but the diffusible nature of both enzyme and dye lowers effective resolution to tissue rather than cell level, and this approach necessarily generates a knockout of the isoform in question, potentially confounding specific functional roles of the isoform.

*In-situ* hybridisation (ISH) enables study of mRNA at the histological level. High-throughput studies typically suffer the same limitations as microarrays (the mouse transcriptome atlas ^37^ uses 3’ sequence only), but 5’ probes have been used to study dp427 expression in embryogenesis ^38^, and more nuanced efforts using first exon sequence-targeted probes allowed Gorecki *et al.* to reveal spatially-distinct dp427 isoforms in the brain ^6, 39^, and Blake *et al.* to study dp71 ^40^. Indeed, expression of dp140 in the developing kidney by Durbeej *et al.* ^18^ was first detected by ISH. Conventional ISH methods are non-optimal, however: enzyme-linked colorimetric methods provide signal amplification, offering sensitivity (at the expense of much quantitative interpretation), but these methods lose significant spatial clarity due to dye diffusion. Radiolabelled probes conversely suffer from low signal to noise ratios and thus tend only to reveal regions of robust expression, missing more subtle expression patterns. Most restrictive of all, ISH methods are typically single-label, permitting only a single target to be discriminated in any given section.

### Dystrophin multiplex ISH

RNAscope ^41^ is a novel ISH method with single-transcript sensitivity and resolution: the approach uses ‘ZZ’ pairs, short oligonucleotides that when bound to adjacent target sequence, create a platform for preamplifier molecules ( Figure 1D). These provide a foundation for multiple amplifiers, which in turn allow targeting of peroxidases. This ‘amplifier tree’ thus generates very high focal concentrations of enzyme in a probe-specific manner: used in combination with tyramide dyes (which covalently link to vicinal tyrosines in the presence of peroxidase activity) this allows mRNAs to be fluorescently labelled as discrete entities. Employing 15-20 ‘ZZ’ pairs in series allows extremely high specificity (target sequences of ~1000 bases) and remarkable sensitivity: for genes of modest expression, single transcripts are resolved as punctate fluorescent foci (apparent size ~1 μm). A further key strength of RNAscope is multiplexing: by using probe-specific enzyme conjugates and blockers, dyes can be added sequentially, resulting in multiple target mRNA species labelled with distinct fluorophores ( Figure 1E). We have modified this multiplex strategy yet further: even with target sequences 1000 bases in length, the 14 kb of the dp427 transcript permits multiple probes to bind, allowing dystrophin transcripts to be labelled with multiple fluorophores in a sequence-specific manner. We have previously employed this multiplex strategy in healthy and dystrophic skeletal muscle ^42^ using probes to the 5’ and 3’ regions of dp427, regions that, given the length of the transcript and the 16-hour transcription time, are separated both spatially and temporally (5’ sequence is transcribed several hours before 3’ sequence ^3, 43^). In healthy mouse muscle ( Figure 1F), punctate sarcoplasmic fluorescent foci of both 5’ and 3’ probes are found in close proximity, indicating dual-labelling of mature, exported dp427m transcripts, while the 5’ probe also produces broad and intensely fluorescent foci within nuclei, indicating the presence of many nascent dp427 mRNAs (20-40 per myonucleus ^42^) arrayed along the dystrophin locus (correspondingly, small nuclear 3’ foci are observed infrequently). In dystrophic *mdx* muscle ( Figure 1G) sarcoplasmic foci (5’ and 3’) are dramatically reduced as expected: mature dp427 transcripts are rapidly degraded by nonsense-mediated decay (NMD), a process that occurs after nuclear export ^44^. Myonuclei can, however, still be identified: 5’ nuclear foci are reduced in intensity (suggesting fewer nascent transcripts, i.e. a reduction in transcriptional initiation), but remain prominent. Dystrophic muscle also reveals rare nuclei associated with 3’ probe labelling only, consistent with dp71 expression in mononuclear cells such as endothelia or proliferating myoblasts. The success of this single-transcript duplex-labelling strategy in revealing both dp427 mRNA dynamics, and distinguishing dp71 from full-length transcripts, suggested that addition of a further ‘middle’ probe (triplex labelling) might permit expression of multiple dystrophin isoforms to be distinguished histologically (see Figure 1A). This approach is described in this manuscript: our 5’ probe recognises exons 2-10 of the full-length dystrophin transcript (dp427). Dp427m, c, and p differ only in their first exon, thus all three dp427 sequences will be detected by this probe set, but all other isoforms of dystrophin will not. Our new middle probe recognises exons 45-55, a sequence present in dp427, dp260 and dp140, but not dp116 or dp71. Finally, our 3’ probe recognises exons 64-75 of the dp427 sequence, and is thus capable of detecting every dystrophin isoform, though it cannot distinguish one from another. Used in combination, these three probes allow a high degree of isoform discrimination within a single sample: only dp427 will label with all three probes, thus presence of 5’ probe indicates full-length dystrophin expression. Given the ~16 hour transcription time, concerted expression of dp427 will result in many nascent transcripts as shown previously ^42^: 5’ signal will be predominantly in the form of large nuclear foci, accompanied by progressively smaller foci of middle and 3’ probes ( Figure 1H). Presence of middle and 3’ probes in the absence of 5’ probe ( Figure 1I) indicates dp260 or dp140, with middle probe likely to form smaller nuclear foci (commensurate with the predicted ~9.5- and ~8-hour transcription times of dp260 and dp140, respectively, assuming consistent transcription at ~40 bases.sec ^-1^ ^45^). Presence of 3’ probe alone ( Figure 1J) thus indicates expression of dp116, dp71, or dp40. As dp116 is believed to be restricted to Schwann cells ^11^, and dp40 lacks almost half of the 3’ probe target sequence (and is expressed at lower levels than dp71 ^16^), most 3’ probe signal is likely to represent dp71. Transcription of dp71 requires ~1 hour, with the full 3’ probe target sequence emerging only ~20 minutes before transcript completion: most dp71 labelling will therefore be in the form of small, single transcript foci.

To demonstrate the utility of this approach, we have used these probes in healthy and dystrophic canine embryos (collected from our deltaE50-MD dog colony ^46, 47^). Our data reveal remarkable diversity in dystrophin isoform expression across the developing embryo and show this gene to be expressed in a wider range of tissues than previously recognised. We further show that isoforms are tightly coupled to distinct tissue subtypes even within defined tissues, providing insight into their developmental roles.

## Methods

### Probe design

20ZZ RNAscope probes (ACDBio) were designed to mouse dystrophin sequence (accession number NM_007868.6). The catalogue probe (Mm-Dmd, Cat. No. 452801) in the C1 channel recognises residues 320-1295 (exons 2-10) of the full-length (dp427) dystrophin transcript, while the custom probes in the C2 (Mm-Dmd-O1-C2, Cat. No. 529881-C2) and C3 channels (Mm-Dmd-O2-C3, Cat. No. 561551-C3) recognise residues 9581-10846 (exons 64-75) and residues 6692-7764 (exons 45-51) respectively. C1 (5’ probe) labels full-length dystrophin isoforms only (dp427c, dp427m, dp427p), C2 (3’ probe) labels all dystrophin isoforms, while C3 (mid probe) labels dp427, dp260 and dp140, but no shorter isoforms (see Figure 1A). Dystrophin sequence is highly conserved (particularly within exons) and the regions covered by these 20ZZ probes show a high identity between mouse and dog (89.27%, 86.39% and 93.52% identical for 5’, middle and 3’ probes, respectively). Positive control probes to POLR2A (NM_009089.2, residues 2802-3678), PPIB (NM_011149.2, residues 98-856) and UBC (NM_019639.4, residues 36-860) were used to confirm preservation of sample RNA, while negative control probes to bacterial DapB (EF191515, residues 414-862) were used to examine possible non-specific labelling ^48^.

### ARRIVE statement

***Animal husbandry***. Dogs were housed at the Royal Veterinary College, in a dedicated canine facility with large pens, daily human interaction and access to outdoor runs and grass paddocks: conditions that exceed the minimum stipulated by the UK, Animal (Scientific Procedures) Act 1987 and according to local Animal Welfare Ethical Review Board approval. Carrier female Beagle (RCC strain)-cross (F3 generation) dogs derived from an original founder Bichon-Frise cross Cavalier King Charles Spaniel female carrier ^46, 47^ were mated with male Beagles (RCC strain) to produce offspring (wild type, carrier and deltaE50-MD). Adult dogs were group housed (12 hour light/dark cycle; 15-24°C) until females were close to whelping; thereafter, pregnant females (singly housed) were allowed to whelp naturally and all puppies within a litter (including those on trials) were kept with their mother in a large pen, to enable nursing with access to a bed under a heat lamp (~28°C). From 4 weeks of age, puppies were also allowed puppy feed (Burns) (ad lib) until weaning at 12 weeks, whereupon dogs not required for studies were rehomed. Dogs over the age of 12 weeks received 2 feeds daily and ad lib water. All animals follow a comprehensive socialisation programme and are acclimatised to routine procedures. Welfare assessments are conducted twice daily.

***Sample numbers***. Canine skeletal muscle samples (~0.5 cm ^3^) were biopsied from 3- and 15-month-old WT and deltaE50-MD dogs by open approach, from the left *vastus lateralis* muscle, with dogs under general anaesthesia (see below) as a component of an ongoing natural history trial (Wellcome Trust grant 101550). Biopsy samples were collected from a total of 18 male dogs.

For RNAscope analysis, 3-month samples, WT N=2; delta E50-MD N=3. 15-month samples, WT N=2; deltaE50-MD N=2. For RNA extraction/qPCR analysis, paired 3-month and 15-month samples were used (repeat sampling from the same animals at the appropriate ages): WT N=7; deltaE50 N=6. Four 15-month qPCR samples (2 WT, 2 deltaE50-MD) were prepared from the same samples used for RNAscope labelling.

A total of 5 canine embryos (1 WT female, 1 WT male, 3 deltaE50-MD males, at gestational age 31 of the canine 63-day gestation) were collected during routine ovariohysterectomy of a single pregnant carrier female from the deltaE50-MD colony (performed for unrelated health reasons), prior to her rehoming. The resected uterus and its embryos were held on ice for 15 minutes, after which it was dissected and prepared as detailed below.

Kidney tissue was collected post-mortem from a single (stillborn at term) WT male pup.

***Anaesthesia***. For muscle biopsy sample collection, animals were administered IV premedication (methadone 0.2mg/kg, medetomidine 1µg/kg) 30mins prior to induction, induced with propofol (to effect: 1–4mg/kg) and maintained under sevoflurane. Animals were administered postoperative carprofen (2mg/kg) analgesia for three days following muscle biopsy.

For ovariohysterectomy, premedication used acepromazine (0.01mg/kg) and methadone (0.1mg/kg), followed by propofol induction and sevoflurane maintenance as above.

### Sample preparation

***Canine skeletal muscle***. Canine muscle biopsy samples were collected as described in the ARRIVE statement, above. Muscle samples were mounted in cryoMbed (Bright instruments Ltd) on cork discs and frozen in liquid nitrogen-cooled isopentane. All muscle tissues were stored at -80°C until use.

***Canine embryos***. A total of 5 canine embryos (1 WT female, 1 WT male, 3 deltaE50-MD males) were collected as described above, at day 31 of 63-day gestation (i.e. after most major organogenesis, and equivalent to mouse day ~14.5-15.5 ^49^). Dead embryos were dissected from the uterus and placenta and bisected sagittally: one half of each embryo was fixed in 10% neutral-buffered formalin for 72 hours at 4°C before being processed to paraffin wax in preparation for sectioning. Small pieces of tail tissue were collected for genotyping, and bisected tissue from the head was stored in RNAlater (Fisher) for subsequent RNA isolation (see below). Additional renal tissue collected from a stillborn WT pup (born at term from a different litter) was fixed and embedded as above.

### Sectioning

Frozen muscle tissues were cryosectioned at -25°C to 8-µm thickness using an OTF5000 cryostat (Bright) and mounted on glass slides (SuperFrost, VWR). Serial sections were collected, and slides were dried at -20°C for 1 hour before storage at -80°C until use.

Wax-embedded embryos/tissues were cooled on ice and sectioned at 4µm thickness using a microtome (Leica Biocut), then floated in a waterbath at 48°C and mounted on Superfrost slides. Slides were dried at 37°C overnight and stored at room temperature in sealed containers (with silica gel desiccants as recommended) until use. Three embryo specimens (1 WT male, 2 deltaE50-MD males) with well-preserved tissue morphology and optimal orientation were taken forward for RNAscope labelling. Adjacent serial sections were stained with haematoxylin and eosin for comparative assessment.

### RNAscope slide preparation

***Fresh-frozen canine skeletal muscle***. Preparation of frozen muscle sections for RNAscope necessitates extended fixation times and an additional baking step as described previously ^42^: slides were removed from -80°C freezer and placed immediately into cold (4°C) 10% neutral-buffered formalin, then incubated at 4°C for 1 hour. Slides were dehydrated in graded ethanol series (50%, 70%, 100% x2, 5 mins in each, room temperature) then air-dried and baked at 37°C for 1 hour. Sections were ringed using hydrophobic barrier pen (Immedge, Vector Labs) and then treated with RNAscope hydrogen peroxide (ACDbio) for 15 mins at room temperature to quench endogenous peroxidase activity. After washing twice in PBS, slides were protease treated (RNAscope Protease IV, ACDbio) for 30 mins at room temperature and washed a further two times in PBS before use in RNAscope multiplex assay (see below).

***FFPE canine embryos***. Paraffin-embedded sections were treated according to the RNAscope multiplex fluorescent reagent kit v2 (ACDbio) protocols for FFPE, with target retrieval using the manufacturer’s ‘alternative method’: slides were immersed slowly in target retrieval buffer (held at a gentle boil) for 15 mins, before cooling directly into room temperature distilled water, followed by ethanol dehydration.

### RNAscope multiplex assay

Multiplex assays were performed as suggested by the RNAscope multiplex fluorescent reagent kit v2 (ACDbio) protocols. Probe mixes used were as follows:

- RNAscope 3-plex positive control probe set (320881): POLR2A, PPIB and UBC (C1, C2 and C3 channels, respectively)
- RNAscope 3-plex negative control probe set (320871): Bacterial DapB (in C1, C2 and C3)
- RNAscope mouse dystrophin probe set: Mm-Dmd (452801), Mm-Dmd-O1-C2 (529881-C2) and Mm-Dmd-O2-C3 (561551-C3) (C1, C2 and C3 probes to 5’, 3’ and middle sequence of the dp427 transcript, respectively; see Figure 1A).

After RNAscope labelling, nuclei were stained with DAPI (ACDbio) for 30 sec, or Hoechst (1/2000 dilution in wash buffer, 5 min) and slides were mounted in Prolong Gold Antifade mounting medium (Thermofisher) and allowed to dry overnight (room temperature, protected from light).

Fluorophores were assigned as follows: 5’ probe (C1), TSA-Cy3; middle probe (C3), TSA-Opal520; 3’ probe (C2), TSA-Cy5. Used with the L5, N3 and Y5 filter cubes, this combination of fluorophores exhibited no signal overlap and allowed appropriate exposure times to be selected without fear of aberrant fluorophore detection. Modest tissue autofluorescence was noted in the L5 (Opal520, middle probe) and N3 (Cy3, 5’ probe) channels, primarily within erythrocytes and liver: 5’ and middle probe were assigned to these channels as their strong nuclear labelling and more restricted expression profiles limited interference from autofluorescence.

### Imaging

Individual images were captured using a DM4000B upright microscope with samples illuminated using an EBQ100 light source and A4, L5, N3 and Y5* filter cubes (Leica Microsystems) and an AxioCam MRm monochrome camera controlled through Axiovision software version 4.8.2 (Carl Zeiss Ltd). Objectives used were 5x HC PL FLUOTAR (NA=0.15) and 20x HC PL FLUOTAR PH2 (NA=0.5). Unless otherwise indicated, 20x objectives were used: this magnification was sufficient to resolve discrete spots corresponding to individual transcripts, while retaining adequate depth of focal plane to allow all elements of the tissue section to remain well-focussed. Where used (skeletal muscle samples only), image analysis was conducted using automated ImageJ macros as described previously ^42^. All macros are in .ijm format, written for the Fiji distribution of ImageJ, and are available at the Figshare repository (see *Underlying data* ^50^).

For whole-embryo fluorescence imaging, ~50 serial images were collected using a 5x objective and merged using the pairwise-stitching algorithm of Preibisch *et al* ^51^. Whole embryo brightfield imaging (H&E) was performed via slide-scanning, using a NanozoomerS60 (Hamamatsu) at 20x magnification. For whole brain fluorescence imaging, ~200 serial images were collected at 20x magnification and merged as for whole embryo images, above. ISH images were overlaid onto corresponding H&E images to identify equivalent regions for direct comparison.

Images shown in figures have been resized for display using the scale function of ImageJ and adjusted for clarity using the window/level tool of the same program. Generalised distribution maps were prepared by exporting individual probe channels and adjusting levels as above, followed by use of the mosaic filter (Photoshop) to downsample and emphasise prominent labelling. Appropriately pseudocoloured maps were overlaid onto the DAPI channel (greyscale). Original whole-embryo images (both stitched fluorescence and slide-scanned H&E) are included in the *Extended data* (Supplementary file 1) ^52^. Raw images used for all other figures (and stitched 20x whole brain fluorescence images) are available at the Figshare repository (see *Underlying data* ^53^, ^54^). All ISH fluorescence images are in four-channel .tif format (DAPI, middle probe, 5’ probe and 3’ probe in channels 1-4, respectively) and can be opened using ImageJ or Qpath ^55^ (both open-source). Data files for Nanozoomer images are in the proprietary .ndpi format but can be viewed using Qpath as above, or using the free NDP.view2 software package.

### Genotyping

Genomic DNA extracted from embryonic tail samples (GeneJet genomic DNA isolation kit, Thermofisher) was used to determine genotype via PCR using primers spanning the deltaE50-MD mutation site, followed by Sanger sequencing (GATC biotech). Sex of embryos was determined via PCR for the male-specific *SRY* gene. Primer sequences are provided in Table 1. All trace data from sequencing results are available at the Figshare repository (see *Underlying data* ^56^).

**Table 1.** Primer sequences used in this study. Primer pairs to unique dystrophin isoforms (dp427c,m,p, dp260, dp140, dp116, dp71) and to the middle (exon 44–45) and 3’ end (62–64) of dp427. Primers with identical sequence are shown in bold. Note that both dp140 and dp116 transcripts will contain exons 62–64 of dp427, while dp260 mRNA will contain both exons 44–45 and exons 62–64. All dystrophin qPCR primer pairs span the exon-exon boundaries indicated. Primer pairs to genomic sequence used for genotyping (spanning the deltaE50 mutation site) or for sexing (the Y-chromosomal SRY gene) are also provided.

Target	Primer	Sequence
Dog dp427c exon1-2	Forward	GGCATGATGGAGTGACAGGA
	Reverse	**TCCAAAAGGTCTAGGAGGCG**
Dog dp427m exon1-2	Forward	AAGGCTGCTGAAGTTGGTTG
	Reverse	TCTCTATGTGCTGCTTCCCA
Dog dp427p exon1-2	Forward	CCACCGCAGAATTTGAAATGTC
	Reverse	**TCCAAAAGGTCTAGGAGGCG**
Dog dp260 exon 1-2 ^*^	Forward	TGGTTTGGTCCTGCAGAGAT
	Reverse	TTTCTATCTCCTGGGCCGAC
Dog dp140 exon 1-2 ^†^	Forward	TGCTCTGAACTAAAACCATCCG
	Reverse	CACCGCAGATTCAGGCTTC
Dog dp116 exon 1-2 ^‡^	Forward	GTAGTCCCCGGTTCAAGCT
	Reverse	TGCATCGTCAGAACCTTCCA
Dog dp71 exon 1-2 ^§^	Forward	CGGTTCTGGGAAGCTCACT
	Reverse	CCTTCTGCAGTCTTCGGAGT
Dog dp427 exon 44-45	Forward	GCGGCGGTTTCATTATGATATG
	Reverse	CAACACTTTGCCGCTGTCC
Dog dp427 exon 62-64	Forward	TCCCTGGGAGAGAGCCATC
	Reverse	TCATGGCAGTCCTGTAAGCT
Dog deltaE50 genotype	Forward	AGCTCTGATTGGAAGGTGGT
	Reverse	ACCTCAGTGTTGTGCTTTTGA
Dog genomic *SRY*	Forward	GGACGGACAATTCAACCTCG
	Reverse	GCATTTTCCACTGGTACCCC

*Exon 2 of dp260 is exon 30 of dp427.

†Exon 2 of dp140 is exon 45 of dp427.

‡Exon 2 of dp116 is exon 56 of dp427.

§Exon 2 of dp71 is exon 63 of dp427.

### RNA isolation and qPCR

Skeletal muscle RNA was prepared from *vastus lateralis*muscle: RNA was isolated from sections collected during cryosectioning as described previously ^57, 58^ (WT: N=7; deltaE50-MD: N=6; same animals used for both 3- and 15-month samples). Embryonic cranium tissues stored in RNAlater (see above) were removed and gently dissected to isolate eye and brain tissues. Isolated eyes, brain and residual tissues of the head were homogenised in microfuge tubes using a plastic micropestle (Fisher). All RNA isolations used TRIzol reagent (Invitrogen) or RNABee (Amsbio), and were assessed by nanodrop to determine yield and purity. A total of 800 ng RNA was used to prepare cDNA via RTnanoscript2 (Primerdesign), with reactions subsequently diluted 20-fold in nuclease-free water. qPCRs were performed in 10-µl volumes using 2 μl cDNA (~8 ng per well assuming 1:1 conversion) in a CFX384 Lightcycler using PrecisionPLUS SYBR green qPCR mastermix (Primerdesign).

Skeletal muscle expression was normalised to HPRT1, SDHA and RPL13a (as described previously ^58^). For multiple embryonic tissues, in lieu of a suitably validated reference gene panel, dystrophin expression data was instead normalized to ‘total dystrophin’ (sum of expression of all isoforms) to permit assessment of relative isoform expression changes on a tissue-by-tissue basis. Evaluated this way, total dystrophin expressionwas comparable between brain and residual head tissue, while expression in the eye (all isoforms) was notably lower. No genotype-dependent global differences were detected in dystrophin gene expression in any tissue. All raw Cq data and analysis is available at the Figshare repository (see *Underlying data* ^59^).

***Primers***. Primers to HRPT1, SDHA and RPL13a (reference genes for skeletal muscle) were taken from the *C.familiaris* geNorm kits (Primerdesign), and are proprietary.

All qPCR primers to canine dystrophin are shown in Table 1. Annotation of the Ensembl canine dystrophin locus is incomplete: the unique first exons designating transcriptional start sites of dp427p, dp427c, dp260, dp140, dp116 and dp71 are not mapped. Mouse dystrophin sequence was instead used as a reference to locate equivalent exon matches in the canine sequence, all of which exhibited high sequence identity. Primers were designed using primer3 software and all span one or more introns. All qPCRs included a melt curve, and all primer sets produced a single amplicon.

***Statistical analysis***. Statistical analysis of qPCR data ( *vastus lateralis* only) was performed with repeated measures 2-way ANOVA with Sidak’s multiple comparisons test (GraphPad Prism 8), with significance set at P<0.05.

## Results

### Multiplex ISH reveals robust dp427 expression in dystrophic canine muscle and elevated expression of dp71

Our RNAscope probes are designed to mouse sequence, but dystrophin is highly conserved between species (84–95% target sequence identity between mouse and dog, see methods). Moreover, mouse-targeted positive control probes (Polr2a, Ppib and UBC) label canine tissue (muscle and embryonic) effectively while negative control probes to bacterial DapB do not ( *Extended data*,Supplementary figure 1 ^60^), suggesting the probes are cross-species compatible.

**Figure 2.**
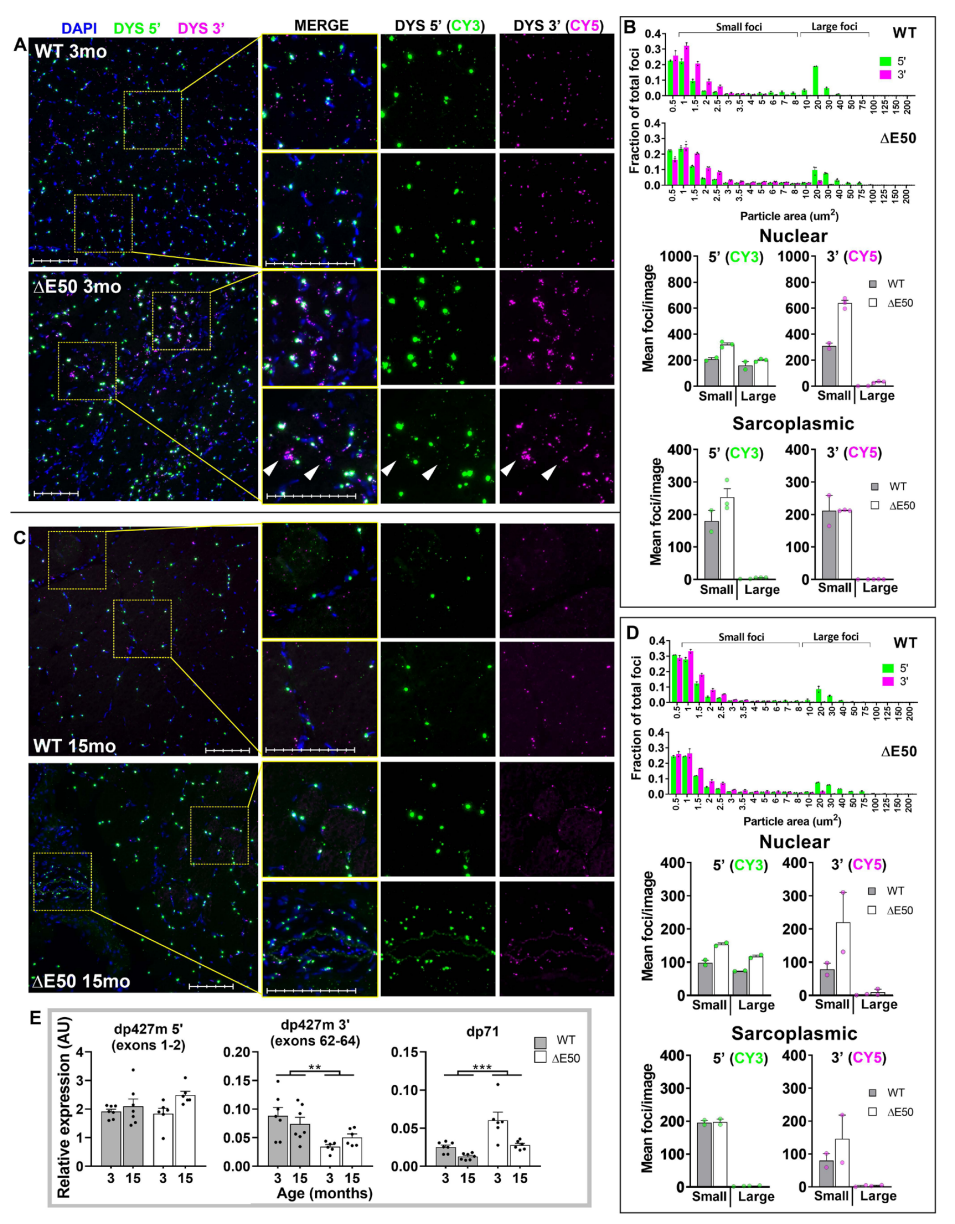
Dystrophin expression in healthy and dystrophic canine muscle. RNAscope probes to mouse dystrophin 5’ and 3’ regions recognise canine dystrophin in both healthy **(WT)** and dystrophic (deltaE50-MD) *vastus lateralis* muscle from 3-month-old ( **A**) and 15-month-old ( **C**) male animals. Nuclei (likely individual cells) associated with many 3’ probe foci are found within dystrophic muscle (arrowheads). Note dystrophin expression in blood vessel smooth muscle ( **C**, deltaE50-MD, lower panels). Apparent size distributions of probe foci ( **B** and **D**, upper panels) and subcellular localisations (lower panels) reveals 5’ foci fall into two populations: one small (~1.5 μm ^2^) and predominately sarcoplasmic; one large (>10 μm ^2^) and exclusively nuclear. 3’ foci are uniformly small (both sarcoplasmic and nuclear). DeltaE50-MD muscle shows no reduction in counts of sarcoplasmic 5’/3’ foci: instead nuclear counts of 3’ foci (commensurate with dp71 expression) tend to be higher. ( **E**) qPCR for 5’ (exons 1-2) and 3’ (exons 62-64) regions of dp427m and for dp71 in 3- and 15-month-old WT and deltaE50-MD *vastus lateralis* muscles, confirms no significant dystrophy-associated reduction in dp427 transcriptional initiation and a modest (~2-fold) reduction in mature transcript number (P=0.006). A concomitant ~2-fold increase in dp71 expression (P=0.0005) is observed. All expression values are arbitrary units, normalised to HPRT1, RPL13a and SDHA (see methods) and adjusted relative to exon 1-2 values for comparative purposes. RNAscope ISH N=2-3 per genotype; qPCR N=6-7 per genotype, repeated measures 2-way ANOVA with Sidak’s multiple comparisons test (WT vs deltaE50-MD). Full-size figure can be found in the *Underlying data* ^35^.

To confirm this, we tested our 5’ and 3’ probes in 3-month-old canine muscle. As shown in Figure 2A, both probes recognize dp427 transcripts expressed by skeletal muscle sampled from healthy dogs (WT), revealing a staining pattern of fluorescent foci comparable to that observed in mouse ^42^. 5’ probe foci fall into two populations: one broad, intense and restricted to nuclei, the other small and punctate, found within nuclei and the sarcoplasm. The 3’ probe foci are instead uniformly small and punctate ( Figure 2B). As described above, this pattern is consistent with high numbers of nascent immature dp427m transcripts within the nucleus, arrayed along the dystrophin genomic locus (large myonuclear foci of 5’ probe only) and mature, exported dp427m mRNAs (small sarcoplasmic foci of both probes, found in close proximity). Unexpectedly, both probes also labelled comparable numbers of dp427m transcripts in dystrophic deltaE50-MD dog muscle, a finding at odds with previous observations in *mdx* mouse muscle, where mature (sarcoplasmic) transcripts are near-absent and nascent nuclear transcript numbers are reduced. To confirm this was not an artefact of youth (dogs at 3 months of age are still growing rapidly) we repeated our labelling in adult muscle samples (15 months, Figure 2C, D), obtaining similar results. Unlike mouse, both young and older dystrophic dog muscle also exhibited markedly greater numbers of 3’ foci than 5’, predominantly within the nuclear compartment, findings confirmed by quantitative image analysis. These 3’ foci were found clustered, apparently restricted to individual nuclei with minimal surrounding cytoplasm and little or no 5’ labelling ( Figure 2A, arrowheads), typically within regions of disrupted muscle architecture.Only rarely observed in mouse muscle ^42^, this pattern is consistent with expression of dp71, likely within proliferating myoblasts ^21^. The deltaE50-MD dog model of DMD exhibits markedly more severe muscle pathological changes than adult *mdx* mice, with widespread areas of focal necrosis and regeneration even at 15 months of age, thus increased labelling of dp71 is not unexpected (marked increases in dp71 expression have also been reported in muscle of young *mdx* mice, i.e. in the acute phase of disease onset ^61^).

To corroborate these findings, we measured transcript levels via qPCR ( Figure 2E), confirming deltaE50-MD muscle shows no reduction in total dp427 transcription (transcripts containing exons 1-2), but does exhibit a ~2-fold reduction in more mature transcripts (exons 62-64). Transcription of deltaE50-MD dp427m thus appears to be initiated at levels comparable with healthy muscle, but is still subject to NMD, albeit to a lesser extent than *mdx* dp427m transcripts. Similarly (again in agreement with RNAscope), deltaE50-MD muscle exhibits a ~2-fold increase in dp71 expression, particularly in younger samples. Our probes thus recognise canine dystrophin sequence, allow different isoforms to be discerned, and moreover reveal differences in transcript behaviour between dystrophic dogs and mice.

### Canine embryos express multiple dystrophin isoforms with clear tissue specificities

**Figure 3.**
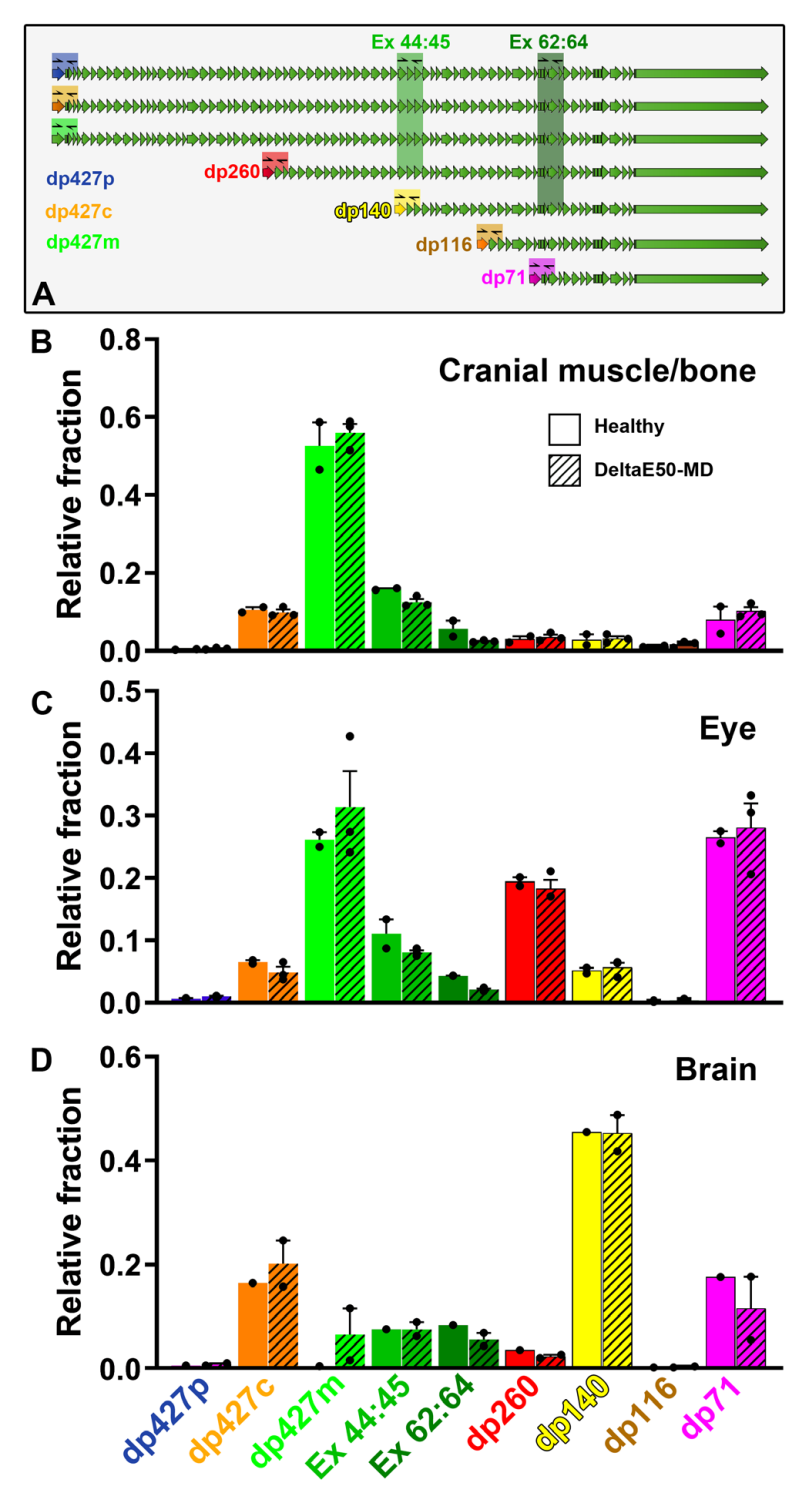
qPCR analysis of dystrophin isoform expression in canine embryos. Locations of primer pairs used ( **A**): forward primers bind to unique first exon sequence. Primers to exons 44-45 and 62-64 will recognise multiple isoforms as indicated (shaded columns). ( **B-D**) Relative expression of dystrophin isoforms (indicated) in embryonic tissues. Cranium muscle/bone tissues ( **B**) predominantly express dp427m, with low levels of dp71 and dp427c. Dp427m is also present in the eye ( **C**) likely in extraocular muscles, but dp71 is prominent in this tissue, as is dp260. Dp140 and dp427c are present at modest levels. In the brain ( **D**) dp140 predominates, with dp71 and dp427c also contributing. Healthy and dystrophic samples reveal comparable patterns of expression for all isoforms, with levels of more mature full-length sequence (exons 44-45 and 62-64) declining progressively toward the 3’ end (a decline more pronounced in deltaE50-MD embryos, consistent with NMD of this transcript). Dp427p and dp116 are detected, but present at only trace levels. Healthy: N=2 (brain N=1); DeltaE50-MD: N=3 (brain N=2). Full-size figure can be found in the *Underlying data* ^35^.

To confirm the presence and relative quantities of different dystrophin isoforms in healthy and dystrophic canine embryonic tissues, we measured expression via qPCR using isoform-specific primers ( Figure 3A) along with those to the exon 44:45 junction (shared by dp427 and dp260) and the region spanning exons 62-64 (shared by all but dp71). Our sample set consisted of RNA isolated from embryonic eye and brain (tissues known to exhibit distinct expression patterns), with the remaining tissues of the head (skeletal muscle, skin and developing bone) as a third pool. As expected, dp427m was the major isoform expressed in this latter pool ( Figure 3B), with minor contributions from dp427c and dp71 (dp116, dp140 and dp260 had only trace expression). In agreement with skeletal muscle (above), levels of nascent dp427 transcript were comparable between healthy and dystrophic samples, and measured expression decreased toward the 3’ end (confirming as expected ^42, 43^ that most full-length transcripts are nascent). In dystrophic samples, levels of 3’ sequence appeared yet lower, again likely reflecting NMD-mediated degradation.

Expression within the eye ( Figure 3C) revealed high levels of dp427m (presumably developing extraocular muscle) and lower amounts of dp427c, and as expected expression of the retinal isoform dp260 was also prominent in this tissue, along with dp71 and to a lesser extent dp140. Expression of 3’ sequence (exons 62-64 of full-length dystrophin) was again somewhat lower in deltaE50-MD samples than in WT, commensurate with NMD of longer isoform transcripts (in the eye this sequence is shared by dp427, dp260 and dp140: dp140 should escape NMD, but represents a minor component of the isoform transcriptional milieu here).

Expression of dystrophin within the brain ( Figure 3D) in contrast revealed marked dp140 expression, with dp71 and dp427c present at lower levels. No clear dystrophic reduction in exon62-64 sequence was noted in this tissue (presumably masked by greater numbers of stable dp140 transcripts).

Beyond the modest reduction in mature full-length transcripts noted above, no deltaE50-MD-specific differences in isoform expression were noted in the tissues studied, suggesting, as shown in mature skeletal muscle, this mutation does not affect transcriptional initiation: multiplex ISH of WT and deltaE50-MD embryos might therefore reveal modest differences in mature dp427 distributions, but nascent transcription of longer dystrophin isoforms (and all dp140 and dp71 mRNAs) should remain unaffected.

Expression of the Schwann cell isoform dp116 was uniformly very low, as was expression of the Purkinje isoform (dp427p). This latter full-length isoform was unambiguously detected in all samples, but expression was orders of magnitude lower than dp427c or dp427m, suggesting that at this embryonic stage, this isoform is either infrequently expressed, or restricted to highly specific (minority) cell populations. Similar results have been reported in human brains ^17^.

### Dystrophin multiplex ISH in canine embryos

**Figure 4.**
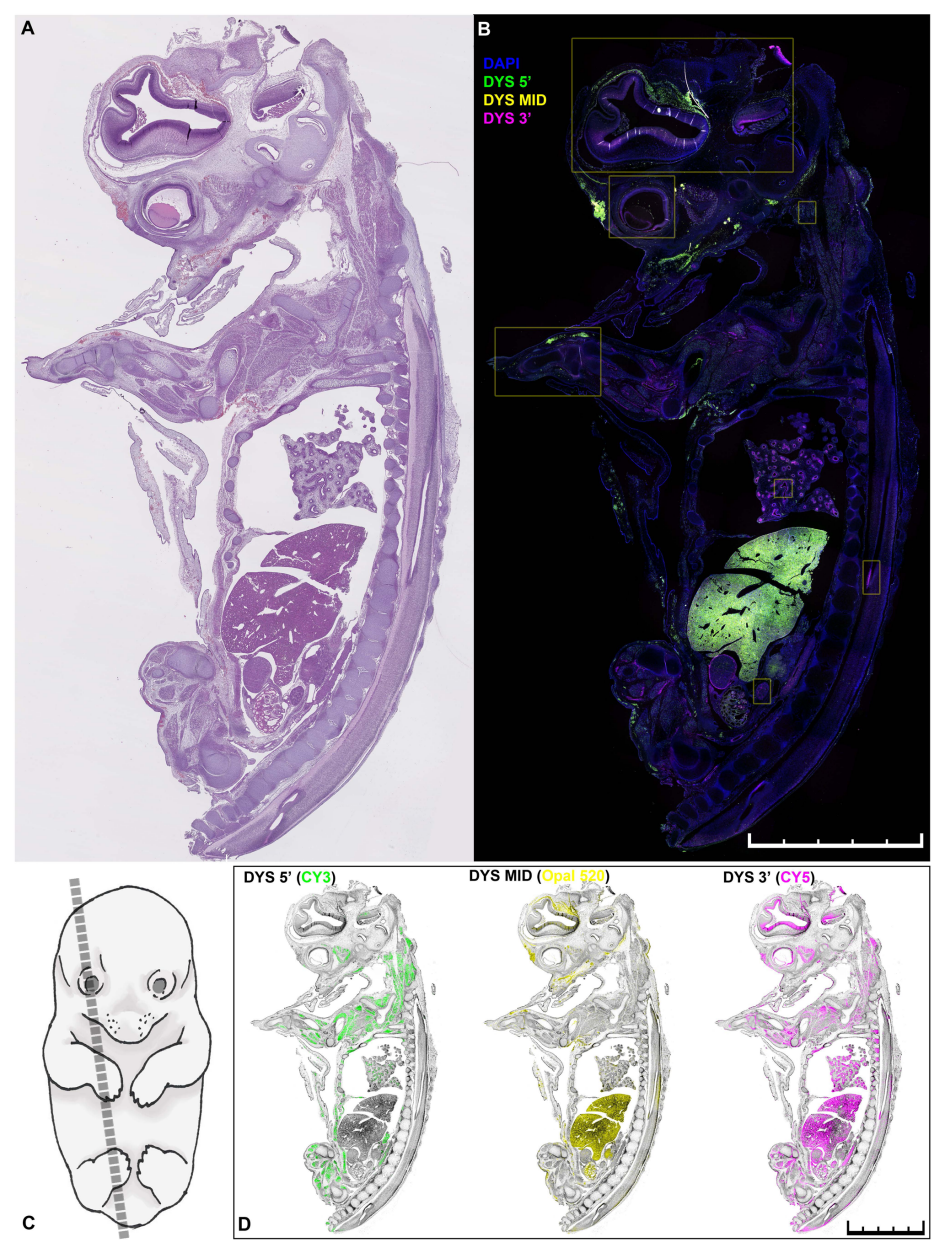
Dystrophin multiplex ISH in canine embryos (deltaE50-MD). Serial sections collected from a deltaE50-MD canine embryo (day 31 of 63-day gestation) stained with haematoxylin and eosin ( **A**) and with dystrophin multiplex ISH probes ( **B**) as indicated (5’ probe: Cy3, green; middle probe: opal 520, yellow; 3’ probe: Cy5, magenta. Nuclei (DAPI): blue). ISH image shown is a composite of ~50 images collected at 5x objective. Regions subsequently examined in greater detail are indicated (brain, eye, neck musculature, forelimb, lung, spine and kidney: yellow boxes). ( **C**) Approximate plane of section for reference. ( **D**) generalised contrast-enhanced distribution map of probe-specific expression: 5’ probe signal is strongest in nascent musculature, lung and cerebellar primordium, middle probe signal in the brain and spinal cord, while 3’ probe signal overlaps with 5’/middle probe, but is also found enriched at joint margins, dorsal root ganglia and major blood vessel walls. Note strong autofluorescence from liver and erythrocytes, particularly in middle probe (opal 520) signal.Scale bars: 5 mm (subdivisions 1 mm). Full-size figure can be found in the *Underlying data* ^35^.

**Figure 5.**
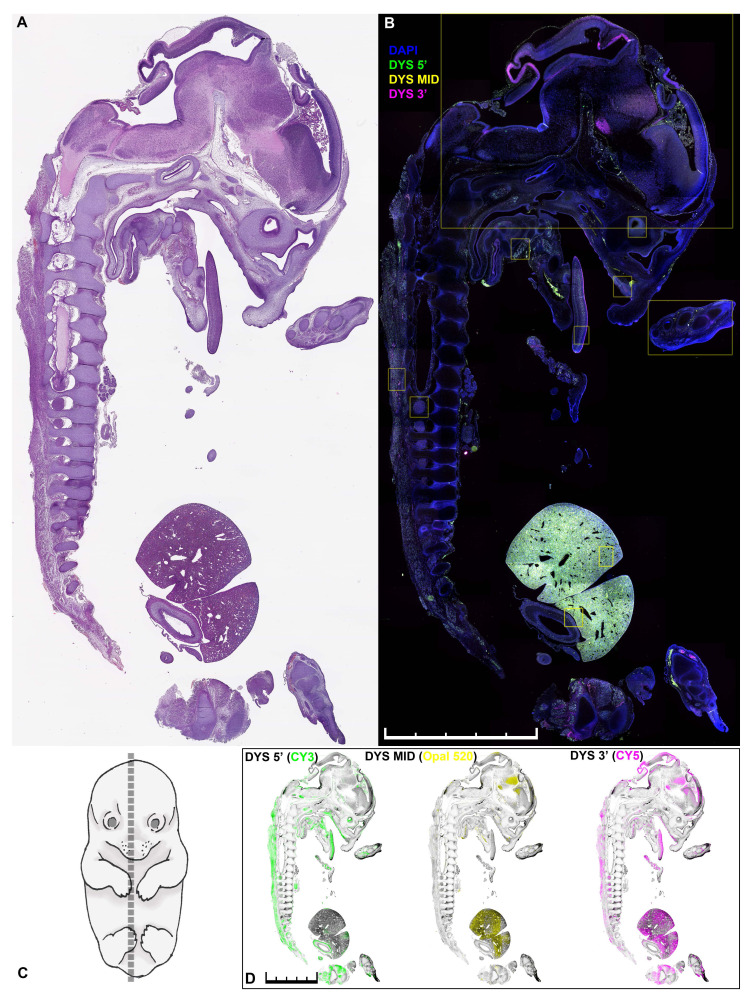
Dystrophin multiplex ISH in canine embryos (WT). Serial sections collected from a healthy WT male canine embryo (day 31of 63-day gestation) stained with haematoxylin and eosin ( **A**) and with dystrophin multiplex ISH probes ( **B**) as indicated (5’ probe: Cy3, green; middle probe: opal 520, yellow; 3’ probe: Cy5, magenta. Nuclei (DAPI): blue). ISH image shown is a composite of ~50 images collected at 5x objective. Regions subsequently examined in greater detail are indicated (brain, neck musculature, vomeronasal organ, dental bud, forelimb, tongue, spinal musculature, dorsal root ganglion, liver, stomach: yellow boxes). ( **C**) Approximate plane of section for reference. ( **D**) Generalised contrast-enhanced distribution map of probe-specific expression: 5’ probe signal is strongest in nascent skeletal and smooth musculature, vomeronasal organ and hindbrain, middle probe signal in the diencephalon/telencephalon, while 3’ probe signal overlaps with 5’/middle probe, but is also found enriched at joint margins, dorsal root ganglia and epithelial linings. Scale bars: 5mm (subdivisions 1 mm). Full-size figure can be found in the *Underlying data* ^35^.

***Multiplex ISH reveals isoform-specific expression patterns***. Whole-embryo ISH with dystrophin multiplex RNAscope probes (5’, middle, 3’) reveals striking probe-specific behaviour, even under low-power (5x) objectives: as shown in Figure 4 and Figure 5 (and *Extended data*, Supplementary figure 2 ^60^), strong nuclear foci of 5’ probe signal (high levels of nascent dp427) are observed in all regions of developing skeletal muscle, and in smooth muscle surrounding larger blood vessels, stomach, and emerging bronchioles of the lung. Prominent nuclear 5’ foci are also found within the developing diencephalon and metencephalon, and in specific (dorsal) sub-regions of the developing spinal cord. Clear nuclear middle probe staining in the absence of any 5’ probe foci (indicative of dp140) was also observed in the brain: central areas of developing diencephalon and telencephalon had widespread middle probe signal, but probe foci were also identified along specific cortical margins, and in ventral domains of the spinal cord. In contrast to the broad nuclear labelling of 5’ and middle-probe sequence, 3’ probe foci appeared small and punctate, but in many places were sufficiently abundant to be resolved at lower magnification. The 3’ probe signal alone (predominantly commensurate with dp71/dp40) was concentrated within endothelial cells of blood vessels, the epithelia of lung bronchioles and nascent skin surfaces, but also within the eye and regions of developing brain.

At this broad morphological level, both healthy ( Figure 5) and deltaE50-MD ( Figure 4) embryos exhibited similar expression patterns. Given the sub-cellular, single-transcript resolution of RNAscope labelling, we examined regions of specific interest at higher magnification.

**Figure 6.**
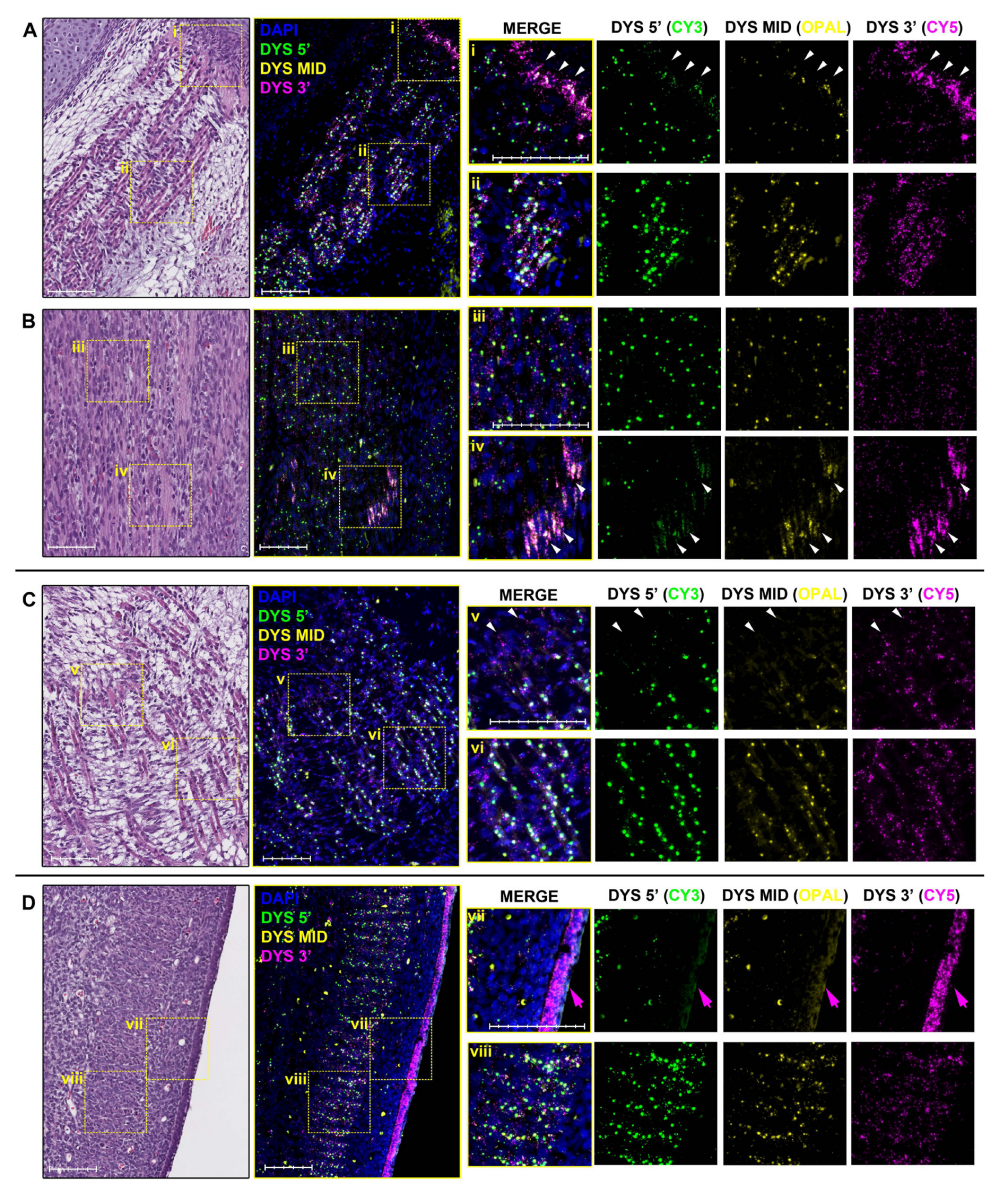
Dystrophin multiplex ISH in healthy and dystrophic canine embryonic muscle. Embryonic primary skeletal muscle fibres from WT ( **A** and **B**) and deltaE50-MD ( **C**) embryos, taken from the neck ( **A** and **C**) and dorsal paraspinal muscles ( **B**) respectively. Haematoxylin and eosin stained serial sections are shown (left panels) alongside equivalent regions probed for ISH. Regions of interest taken for enlarged channel-specific insets (rightmost panels) are indicated. Myonuclei contain large deposits of 5’ probe and middle probe signal, while nuclear 3’ probe signal is less prominent, indicating presence of nascent dp427 transcripts. Small foci of all three probes are found outside myonuclei, consistent with mature exported dp427 mRNAs within the sarcoplasm (correspondingly reduced in deltaE50-MD muscle). Extranuclear signal from all three probes is prominently concentrated at presumptive myotendinous junctions in WT muscle (arrowheads, insets **i** and **iv**), but not deltaE50-MD muscle (inset **v**) where 3’ signal alone is present. ( **D**) Multiplex ISH of the tongue (WT) reveals isoform-specific expression: primary myofibres within the tongue (inset **viii**) label with all three probes similar to other skeletal muscle (above), while developing tongue epithelia (inset **vii**, magenta arrow) labels intensely with 3’ probe alone, consistent with high levels of dp71 expression. Images sections shown in Figure 4 and Figure 5. Scalebars: 100µm. Subdivisions: 20µm (larger panels); 10µm (insets). Full-size figure can be found in the *Underlying data* ^35^.

***Primary skeletal muscle fibres robustly express dp427 and exhibit subcellular mRNA targeting***. Developing muscle at this embryonic stage derives from primary myogenesis: aligned myotubes that will ultimately serve to orient fibres laid down in secondary myogenesis. Muscle dystrophin is expressed at this stage (confirmed here by qPCR: dp427m, Figure 3B), and as shown, whether in the musculature of the neck ( Figure 6A) or of the dorso-lateral paraspinal region ( Figure 6B), dystrophin multiplex ISH in healthy embryonic skeletal muscle reveals robust expression of dp427 similar to that observed in mature muscle. Nuclei lying within primary myotubes were host to large 5’ probe deposits of strong fluorescence intensity, commensurate with high numbers of nascent transcripts within the dystrophin genomic locus. Correspondingly, each nucleus also revealed a middle probe focus of lower intensity, as would be expected with multiplex labelling of nascent transcripts (nascent mRNAs would acquire 5’ probe sequence early in transcription, while middle probe sequence would be present only within those midway through transcription or later). As with mature muscle, minimal nuclear labelling with 3’ probe was observed (3’ sequence arises shortly before transcript completion and would thus rarely be present in nascent transcripts); instead, small 3’ foci, along with small foci of both 5’ and middle probes, were present in modest quantities along the entire length of nascent myotubes, a staining pattern consistent with multiplex-labelled mature dp427m transcripts. Notably, high numbers of these mature transcripts were found 20–40μm distant from dp427-expressing nuclei, concentrated at locations consistent with developing myotendinous junctions (MTJs; see Figure 6A, B, insets i and iv). Dp427 protein is known to be concentrated at the MTJ ^62, 63^ due to the tightly folded, interdigitated nature of the cell membrane at this location: our data suggests that dp427m mRNA might therefore be specifically targeted to sites with uniquely high demands for dystrophin protein. In embryonic deltaE50-MD muscle, prominent nuclear labelling (5’ and middle probe) was still observed as predicted ( Figure 6C), and while small extranuclear foci of all probes were present, numbers were markedly lower. No dp427 mRNA accumulations were observed at any presumptive MTJs in deltaE50-MD muscle ( Figure 6C inset v, and *Extended data*, Supplementary figure 3B ^60^), and these sites might therefore represent a key point of weakness in DMD (as has been suggested ^62^). In contrast, numerous 3’ foci (consistent with dp71 expression), were observed at putative deltaE50-MD MTJ loci, and 3’ signal was also present in healthy MTJs (more than can reasonably be attributed to dp427 alone), suggesting this short isoform might be canonically expressed at the developing MTJ alongside dp427. Myocytes within the developing deltaE50-MD heart also had modest nuclear labelling consistent with dp427 ( *Extended data*, Supplementary figure 3A ^60^).

Prominent differential isoform labelling was identified in tongue tissue ( Figure 6D, healthy): muscle fibres within the body of the tongue were clearly visible (large 5’ and middle probe nuclear foci arrayed along myotubes, with scattered surrounding small foci of all three probes) while cells of the tongue epithelium (expected to express dp71 only) stained richly with 3’ probe alone.

**Figure 7.**
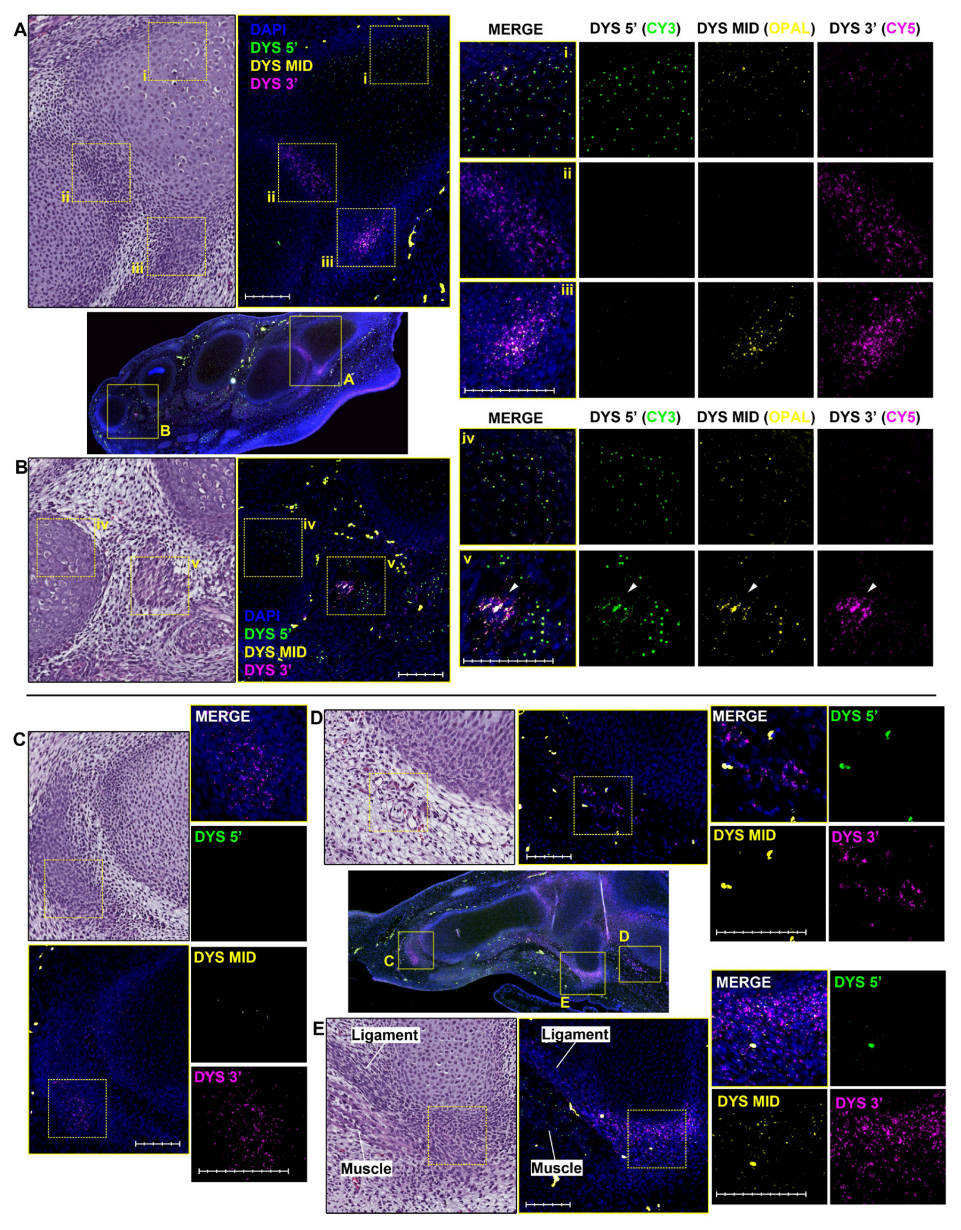
Dystrophin multiplex ISH in healthy and dystrophic canine thoracic limbs. Embryonic thoracic limb from WT ( **A** and **B**) and deltaE50-MD ( **C**, **D** and **E**) embryos. Haematoxylin and eosin stained serial sections are shown alongside equivalent regions probed for ISH. Regions of interest taken for enlarged channel-specific insets are indicated. Labelling of all three probes consistent with dp427 (strong nuclear 5’ and middle probe signal, scattered small 3’ foci) is found within maturing chondrocytes ( **A** and **B**, insets **i** and **iv**), while interzonal mesenchyme labels with 3’ probe alone indicating dp71 ( **A**, inset **ii**). Sites of presumptive entheses show numerous 3’ foci but also label robustly with middle probe, indicating presence of dp140 alongside dp71 ( **A**, inset **iii**: WT; E: deltaE50-MD). Dp140 is similarly found within the distal-most cartilaginous skeletal element, with dp71 more prominently expressed ( **C**, deltaE50-MD). Dp71 is also detected robustly in blood vessel endothelia ( **D**: deltaE50-MD). Areas of skeletal muscle label as expected: prominent nuclear 5’/middle probe foci, strong signal of all three probes at myotendinous junctions ( **B**, inset **v**, arrowheads: WT). Images collected from multiplex-probed sections shown in Figure 4 and Figure 5. Scalebars: 100 µm. Subdivisions: 20 µm (larger panels); 10 µm (insets). Full-size figure can be found in the *Underlying data* ^35^.

***Developing skeletal tissue expresses multiple dystrophin isoforms***. The interplay between muscle and bone during embryonic development is well-established ^64^, but expression of dystrophin within developing bone itself has not been reported. As shown in Figure 7 (A and B: healthy; C, D and E: deltaE50-MD), multiplex labelling in skeletal tissues of embryonic thoracic limb unexpectedly revealed isoform-specific dystrophin expression. Dp427 was found in muscle and enriched at presumptive MTJs as described above ( Figure 7B, inset v, arrowheads), but this isoform was also found within nascent bone, apparently restricted to maturing chondrocytes ( Figure 7A, insets i and iv), implying dp427 could play a role in establishment of skeletal architecture. Cells within the cartilaginous interzone lying between adjacent bones were rich in dp71 (inset ii), as were the endothelial linings of even small blood vessels ( Figure 7D), and cells at the distal growing limb tip ( Figure 7C). Unexpectedly, this latter tissue also exhibited scattered labelling consistent with expression of dp140 alongside dp71, and more pronounced dp140 labelling was found at specific lower margins of more distal bones, restricted to regions that might represent developing entheses of nascent ligament attachment sites ( Figure 7A, inset iii and 7E).

**Figure 8.**
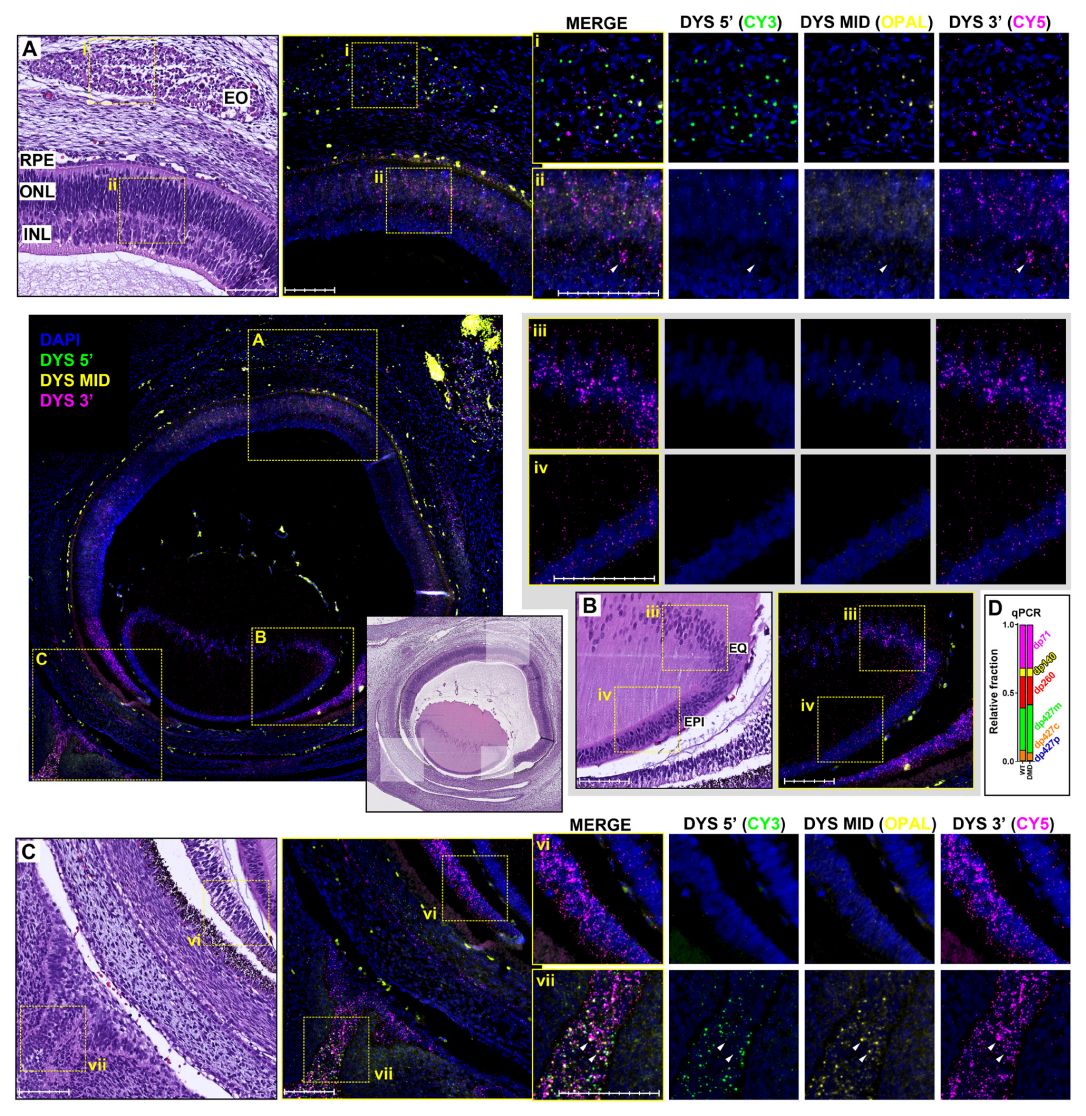
Dystrophin multiplex ISH in the embryonic canine eye. Main panel (centre left): multiplex ISH-probed eye from deltaE50-MD embryo (inset panel: aligned haematoxylin and eosin stained serial section). Regions used to examine retinal/extraocular ( **A**), lens ( **B**) and iris/eyelid ( **C**) ISH are indicated. ( **A**) Extraocular muscles (EO, inset **i**) exhibit the characteristic labelling of dp427, while dp427 expression in the retina (inset **ii**) is more modest and found in only a minor subset of nuclei. Middle/3’ probe labelling corresponding to dp260/dp140 is primarily restricted to the outer neuroblastic layer (ONL) while 3’ probe signal of dp71 is also found at modest levels within the inner neuroblastic layer (INL) and retinal pigmented epithelium (RPE). Isolated cells labelled with many 3’ foci are also found rarely (A, inset **ii**, arrowheads) which may correspond to dp71-expressing endothelia or retinal Müller glia. ( **B**) Nuclei of nascent crystalline fibres in the lens equatorial zone (EQ, inset **iii**) exhibit weak middle probe labelling indicating modest expression of dp140, while 3’ probe signal is more widespread and found through the lens fibres, suggesting dp71 is co-expressed in this tissue. Lens epithelial cells (EPI, inset **iv**) express dp71 only. ( **C**) Ciliary body progenitor cells of the optic cup periphery express high levels of dp71 (inset **vi**) while cells forming the epithelial bridge of the fusing eyelid exhibit robust labelling consistent with multiple isoforms: nuclei labelling with 5’/middle probe foci (dp427) are found in close proximity to those labelling with middle probe alone (dp140, **C** inset **vii**, arrowheads) while high numbers of 3’ foci suggest dp71 may also be present. Images collected from multiplex-probed section shown in Figure 4. Scale bars: 100 µm. Subdivisions: 20 µm (larger panels); 10 µm (insets). Full-size figure can be found in the *Underlying data* ^35^.

***Dystrophin isoforms are differentially expressed across tissues of the developing canine eye***. A number of morphologically distinct structures can be discerned within the developing eye at this stage: as shown in Figure 8, our multiplex ISH approach reveals striking isoform-specific patterns of dystrophin expression within this complex tissue. Nascent extraocular muscles surrounding the globe were clearly resolved ( Figure 8A, inset i), showing strong nuclear 5’ and middle probe foci surrounded by smaller foci of all three probes. Retinal staining, in contrast, had more nuanced patterning ( Figure 8A, inset ii). Sparsely distributed 5’ positive nuclei, less intense than those of muscle, were identified within the developing retinal outer neuroblastic layer (ONL), frequently, but not invariably, accompanied by concomitant middle probe staining. Given qPCR detection of dp427c, these might reflect stochastic ‘bursting’ of dp427c expression (brief, infrequent transcriptional initiation events), rather than the sustained output of muscle dp427m: within the first ~8 hours of transcriptional initiation, 5’ probe sequence will be present within nascent transcripts but middle probe sequence will not. Both ONL and inner neuroblastic layer (INL) of the retina exhibited high numbers of small middle and 3’ foci (most prominently in the ONL), presumably reflecting expression of the retinal isoform dp260: our probe strategy cannot distinguish dp260 from dp140, but qPCR ( Figure 8D) confirmed dp260 is more prominently expressed in the eye, and this isoform is associated with synaptic maturation within the retinal ONL ^65^. Dp71 (small 3’ probe foci alone) was present throughout the retina, but distributed non-uniformly: single nuclei rich in 3’ probe alone were found within the INL, possibly demarking Müller glial cells ^66^, and dp71 expression increased at the optic cup margins ( Figure 8C, inset vi) where the non-neuronal cells that form the ciliary body progenitor pool reside ^67^. Dystrophin transcripts were also detected within the developing lens ( Figure 8B): 5’ foci were absent, but mid-probe and 3’ foci were found within and immediately surrounding the equatorial nuclei of primary lens fibres, indicating expression of dp140 (dp260 is not found within the lens ^68^). 3’ foci (dp71) were also dispersed throughout the lens fibres themselves, and the developing lens epithelium also prominently expressed this short isoform.

Unexpectedly, robust labelling of all three probes was found specifically within epithelial cells lying along the margin of eyelid fusion ( Figure 8C, inset vii), indicating that this population of non-muscle cells expresses dp427 and, given the clear presence of middle-probe nuclear foci free of 5’ probe (arrowheads), likely dp260/dp140 as well. Formation of this epithelial bridge is known to involve concerted cell movement under tension (as the leading edges of the fusion site ‘tow’ the eyelids toward each other), mediated principally via actomyosin cables in a manner similar to smooth muscle contraction ^69, 70^: our data suggests that such specialised movement is accompanied by similarly specialised expression of dystrophin.

**Figure 9.**
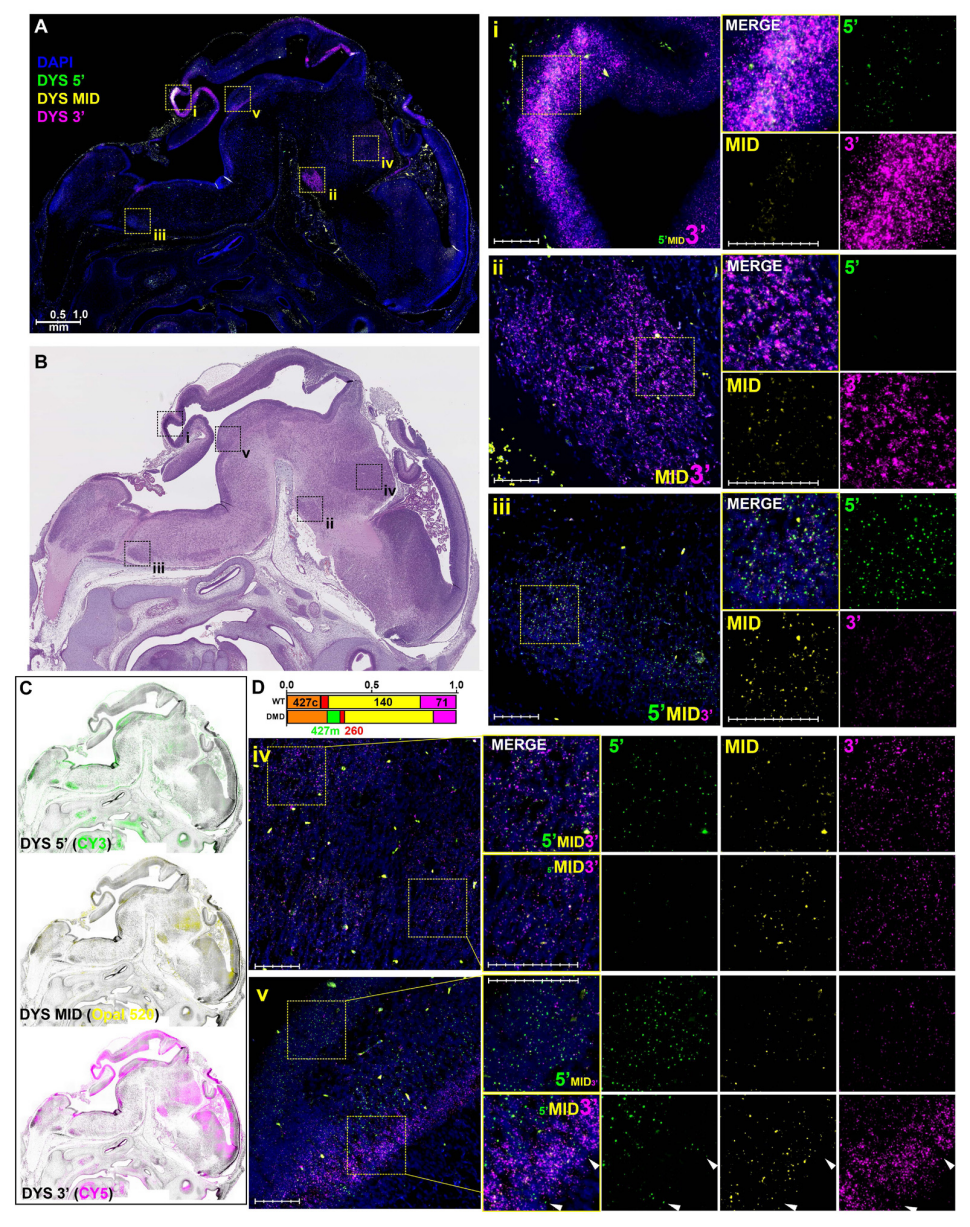
Dystrophin multiplex ISH in the developing WT canine brain. Multiplex ISH probed brain in parasagittal section close to the midline, from a WT embryo ( **A**) and matching haematoxylin/eosin stained serial section ( **B**). Regions studied in greater detail are indicated ( **i**– **v**), with relative levels of probe signal denoted by font size (of appropriate probe and colour). ( **C**) Generalised contrast-enhanced distribution map of probe-specific expression: 5’ probe signal is most prominent in deeper regions of the developing hindbrain, and along specific periventricular regions such as the midbrain tegmentum and prepontine isthmus. Middle probe signal predominates in the primordial thalamus, hypothalamus and caudal telencephalon, while 3’ probe signal overlaps with 5’/middle probe, but is also enriched along all germinal layers, particularly in dorsal developing mesencephalon. ( **D**) Relative expression of dystrophin isoforms in WT and deltaE50-MD embryonic brain via qPCR (taken from Figure 3, provided for reference) showing that dp427c, dp140 and dp71 are the major isoforms. Magnified regions: ( **i**) the developing midbrain roof surfaces marking the transition from mesencephalon to metencephalon express very high levels of dp71, with trace expression of dp427 (5’, middle and 3’ probe) also detected. ( **ii**) Full-length dystrophin is not found in the developing hypothalamus, but middle probe signal consistent with dp140 is present at modest levels alongside marked 3’ signal suggesting co-expression of dp71. ( **iii**) Nuclei deeper within the developing myelencephalon exhibit labelling consistent with expression of dp427 alone. ( **iv**) Dystrophin isoforms are spatially segregated in the developing thalamus: caudal regions express modest levels of dp427 (upper insets), while more rostrally dp140 is found (lower insets). ( **v**) Dystrophin isoforms are spatially segregated at the developing tegmentum/prepontine isthmus boundary: nuclei lying along the germinal layer of the tegmentum express modest levels of dp427 (upper insets), while at the boundary demarking the prepontine isthmus labelling switches abruptly to a pattern consistent with dp140 and dp71 (lower insets), though interdigitating cells expressing dp427 (conspicuous by concomitant reduced 3’ labelling) are also found (lower insets, arrowheads). Images collected from multiplex-probed section shown in Figure 5. Scalebars: main panel: 1 mm. Other panels: 100 µm. Subdivisions: 20 µm (panels **i**– **v**); 10 µm (insets). Full-size figure can be found in the *Underlying data* ^35^.

**Figure 10.**
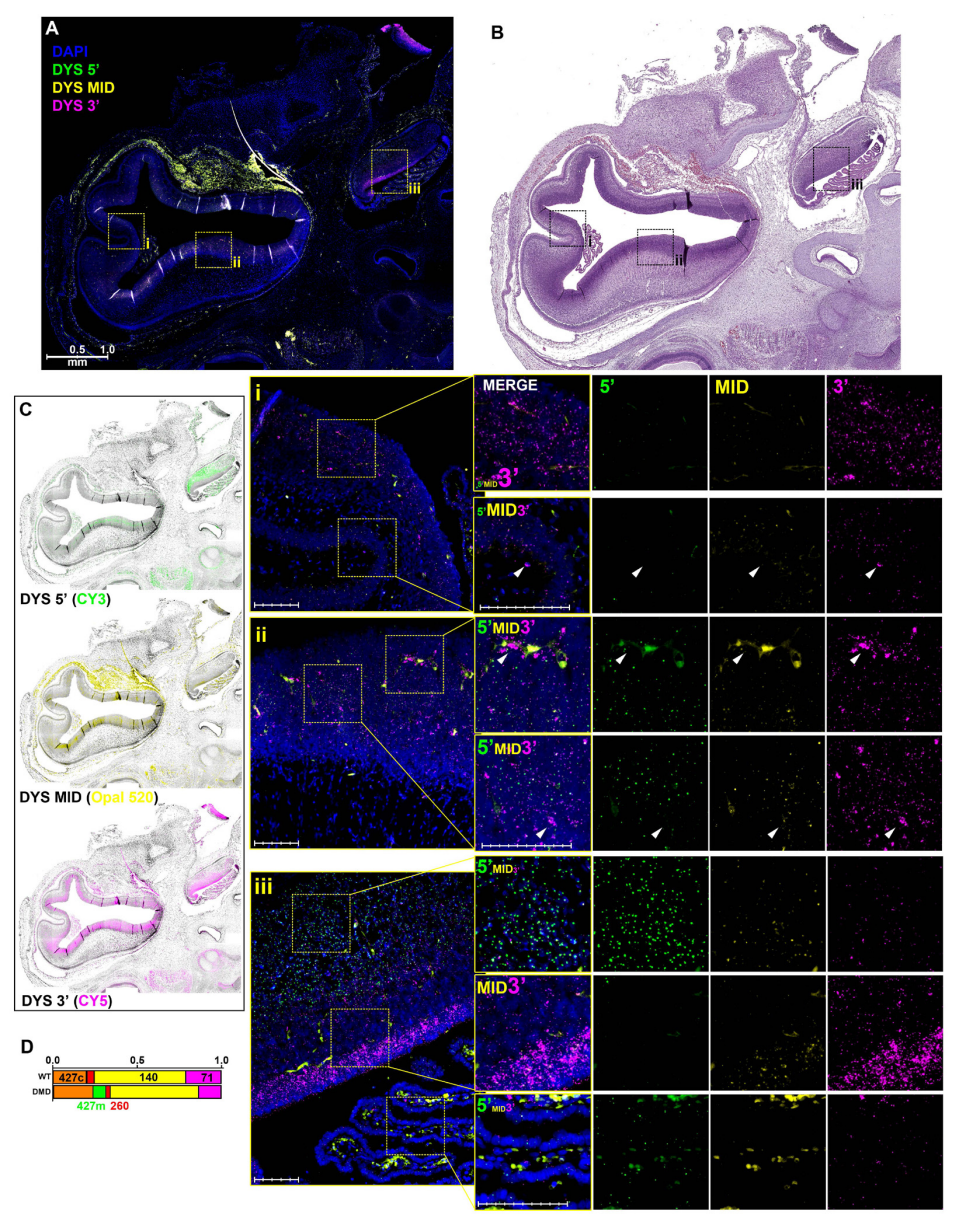
Dystrophin multiplex ISH in the developing deltaE50-MD canine brain. Multiplex ISH probed brain in rightward lateral section from a deltaE50-MD embryo ( **A**) and matching haematoxylin/eosin stained serial section ( **B**). Regions studied in greater detail are indicated ( **i**– **iii**), with relative levels of probe signal denoted by font size (of appropriate probe and colour). ( **C**) Generalised contrast-enhanced distribution map of probe-specific expression: 5’ probe signal is most prominent in the ventral cerebellar primordium and within the germinal layer of the developing telencephalon. Middle probe signal is chiefly concentrated along the germinal layers of the cerebellar primordium and the mantle of the developing telencephalon, while 3’ probe signal overlaps with 5’/middle probe but is particularly enriched along periventricular borders of germinal layers. ( **D**) Relative expression of dystrophin isoforms in WT and deltaE50-MD embryonic brain via qPCR (taken from Figure 3, provided for reference) showing that dp427c, dp140 and dp71 are the major isoforms. Magnified regions: ( **i**) the germinal layer of the developing telencephalon chiefly expresses dp71 (upper panels), while sparse dp140 is found within the developing cortical plate (lower panels) along with rare dp71 expressing cells (arrowheads). ( **ii**) Ventral cortical regions show modest expression of dp427 along with dp71-rich endothelia and glia (arrowheads). ( **iii**) The rhombic lip (cerebellar primordium) expresses a gradient of dystrophin isoforms: cells within the marginal layer express dp427 (upper panels) while cells of the germinal layer express dp140 and dp71, with dp71 alone found immediately proximal to the ventricular space (middle panels). The developing choroid plexus exhibits labelling consistent with very modest expression of dp427 (lower panels). Images collected from multiplex-probed section shown in Figure 4. Scalebars: main panel: 1 mm. Other panels: 100 µm. Subdivisions: 20 µm (panels **i**– **v**); 10 µm (insets). Full-size figure can be found in the *Underlying data* ^35^.

***Embryonic brain exhibits complex, spatially separated patterns of dystrophin isoform expression***. Neural tissues undergo dramatic growth, differentiation and specialisation throughout embryogenesis: at the stage shown here many features of the adult brain are emerging. qPCR data indicated that dp140, dp71 and dp427c are all robustly expressed in the developing canine brain, with dp140 the most abundant isoform (as also suggested by others ^10, 17^). Consistent with this, as shown in Figure 9 and Figure 10, foci of all three probes were identified in the developing brain, however respective extents of probe labelling varied markedly across this tissue, suggesting focally varied expression. In sections closest to the midline ( Figure 9, magnified from Figure 5, WT), pronounced but focal expression of dp71 was found within the cell-rich cranial and caudal poles of the developing mesencephalon roof plate, particularly caudally ( Figure 9 i) where the dorsocaudal mesencephalon folds ventrally to demark the boundary with the rhombic lip (cerebellar primordium). Scant 5’ and middle probe foci were however also observed, indicating that while dp71 might predominate, dp427 is also present. Similarly, the developing diencephalon ( Figure 9 ii and iv) exhibited probe labelling consistent with dp140 (strong middle and 3’ probe, without 5’ probe signal). Ventrally (within the developing hypothalamus) the numbers of 3’ foci appeared higher than could be accounted for by dp140 alone (compare region ii with region iv lower panels), suggesting that this region is host to both dp140 and dp71 (indeed dp71 is known to be highly expressed in specific subregions of the developing brain ^19^). Dp427 expression was prominent in the developing myelencephalon ( Figure 9 iii), particularly ventrally, and within the developing diencephalon ( Figure 9 iv, upper panels), alongside, but spatially distinct from, the dp140 expressing cells nearer the hemispheric sulcus (lower panels). Such restricted, localised isoform expression was also found at the boundary of the developing midbrain tegmentum and the prepontine isthmus, proximal to the mesencephalic aqueduct ( Figure 9 region v): modest dp427 expression was found within the germinal zone (region v, upper panels) and high levels were detected throughout the intermediate zone beneath. At the developing cell-dense fold demarking the tegmentum/isthmus boundary itself (region v, lower panels), expression altered abruptly: a few sparse cells retained small nuclear 5’ and middle probe foci consistent with dp427 (arrowheads, notable also for the concomitantly modest numbers 3’ foci within these cells), but the bulk expression pattern was more consistent with dp140 and dp71.

In parasagittal section ( Figure 10, magnified from Figure 4, deltaE50-MD) expression of dystrophin varied within cortical regions surrounding the developing right lateral ventricle. Rostrally, dp71 predominated in the cells of the germinal zone, while the folding surfaces of the developing telencephalic cortical plate expressed dp140 ( Figure 10 i). More caudally, modest and essentially uniform dp427 expression was observed within the subventricular zone ( Figure 10 ii), though sparse single cells expressing high levels of dp71 were also present (arrowheads). Proximity to blood vessels suggests some of these cells are endothelia (10 ii, upper panels) but others might represent cortical glia (glial cells are known to express this short isoform ^66, 71^). Low levels of dp427 were also identified in the developing cerebellar choroid plexus ( Figure 10 iii, lower panels), but this transcript was expressed at much higher levels (comparable to skeletal muscle) in the deep neuroblasts of the cerebellar primordium ( Figure 10 iii, upper panels). Remarkably, dp427 transcription ceased sharply at the mantle zone where only scant 3’ foci denoting modest dp71 expression were observed, before changing once more to reveal profound expression of dp140 and dp71 within the proliferating ventricular zone (region iii, middle panels—note also that the cell layer most proximal to the ventricular space expresses dp71 alone). This distinctive pattern of variable isoform expression was confirmed bilaterally ( *Extended data*, Supplementary figure 4 ^60^).

**Figure 11.**
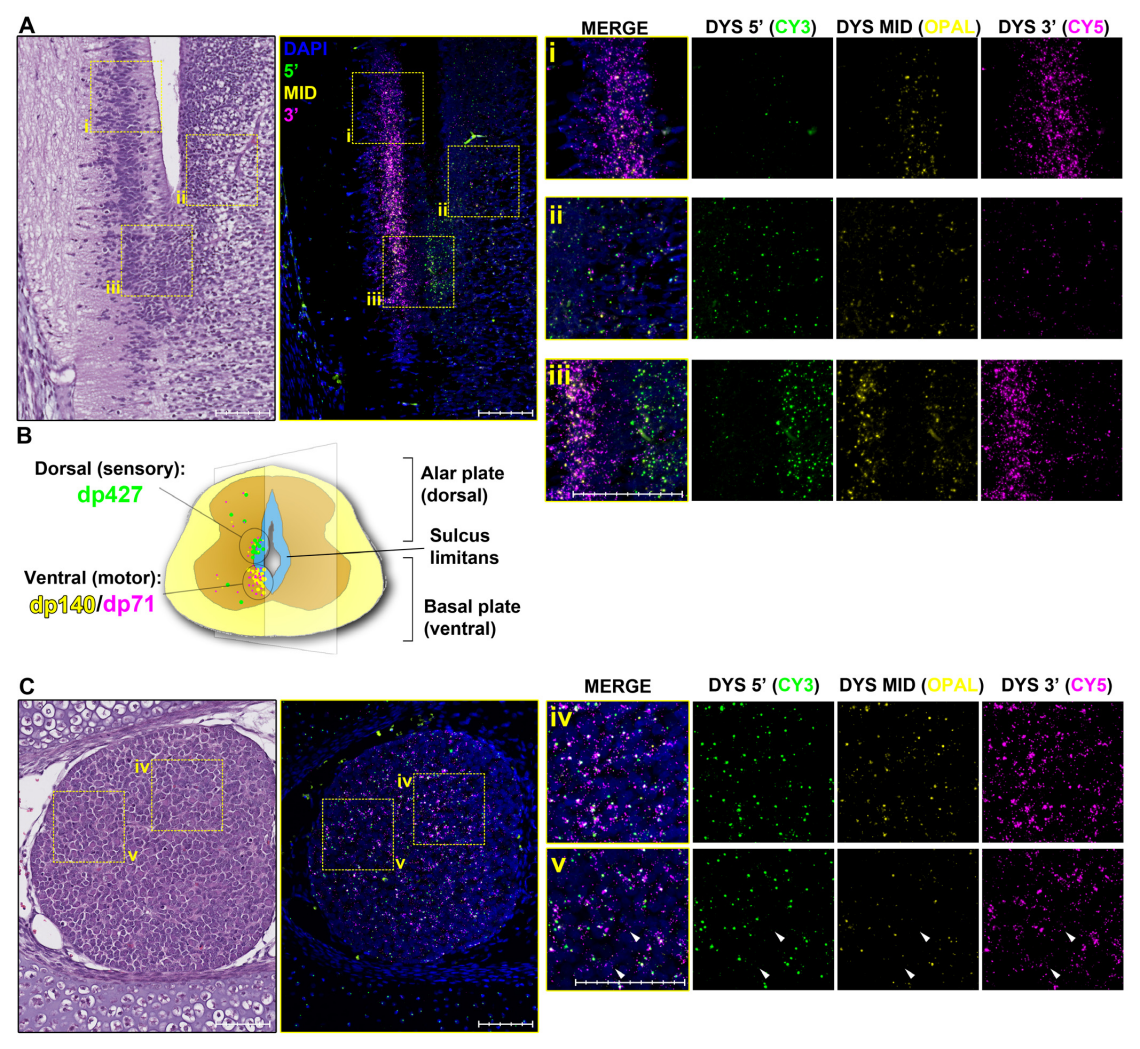
Dystrophin multiplex ISH in the developing canine spinal cord. ( **A**) Dystrophin isoform expression in the spinal cord of a deltaE50-MD embryo (right panel), with aligned haematoxylin/eosin stained serial section (left panel). Ventral (basal plate) regions of the developing spinal cord are rich in dp140 and dp71 ( **i**) while dorsal (alar plate) regions almost exclusively express dp427 ( **ii**). This strict dorso-ventral segregation is defined sharply by the sulcus limitans ( **iii**). ( **B**) Schematic of proposed dystrophin expression in transverse section, with ventral regions expressing dp140/dp71 while dorsal regions express dp427 (approximate plane of section shown in **A** is shown). ( **C**) Dorsal root ganglia from a WT embryo: haematoxylin and eosin (left panel) and dystrophin multiplex ISH (right panel). Labelling is consistent with modest but widespread neural expression of dp427 interspersed with dp71 expressing dorsal root glia ( **iv** and **v**, arrowheads). Images collected from multiplex-probed sections shown in Figure 4 and Figure 5. Scalebars: 100 µm. Subdivisions: 20 µm (panels **i**– **v**); 10 µm (insets). Full-size figure can be found in the *Underlying data* ^35^.

***Dp140 and dp427 are dorso-ventrally segregated in developing spinal cord***. The intricate structure of the developing spinal cord is challenging to interpret fully under sagittal sectioning (see Figure 4 and Figure 5). Small numbers of 3’ foci (dp71) were found in the germinal layer immediately surrounding the central canal, while the characteristic three-probe staining of dp427 expression was identified sporadically throughout the mantle zones (precursors to the dorsal/ventral horns), interspersed with rare but strongly dp71-positive cells. A striking exception to this pattern was found as the plane of section crosses the central canal ( Figure 11A): here dystrophin expression was markedly elevated, with strong expression of dp140 and dp71 within the ventral basal plate ( Figure 11A region i) contrasted with expression of dp427 in the dorsal alar plate ( Figure 11A region ii), a delineation defined sharply by the sulcus limitans ( Figure 11A region iii). Dorso-ventral patterning of the neural tube begins early in development ^72^, and marked dorso-ventral restriction of spinal cord expression is found in other genes, including Hox-2 ^73^, pax3/6 and bHLH factors mash1/neurogenin ^74^. Isoform-specific partitioning of the same gene (as shown here) suggests sensory/motor specializations: the dorsal/alar zones of the spinal cord (dp427) are primarily associated with afferent sensory signalling, while the ventral/basal zones (dp140/dp71) mediate efferent motor signals ( Figure 11B) ^72, 74^. Within dorsal root ganglia ( Figure 11C) expression was both more uniform and more complex: the three-probe labelling of dp427 was found in many nuclei (likely neural crest-derived neuron cell bodies) distributed throughout the ganglion, but close inspection suggested a second cell population expressing dp71 only ( Figure 11C region v, arrowheads), perhaps proliferating neural progenitors, or possibly developing glial satellite cells ^75^.

**Figure 12.**
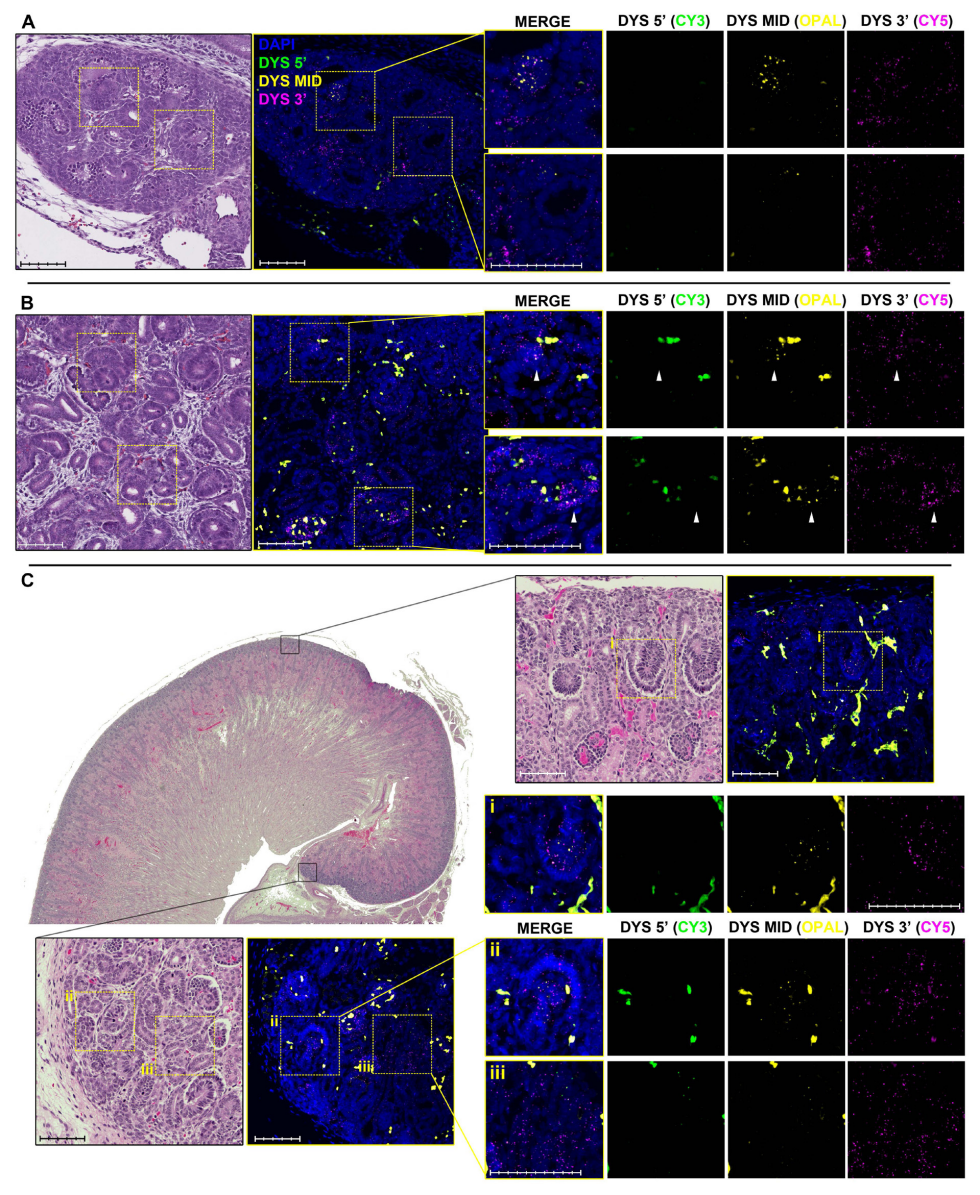
Dystrophin multiplex ISH in the developing kidney. Developing kidneys from deltaE50-MD embryos ( **A** and **B**) exhibit labelling consistent with dp140 expression restricted exclusively to the convoluted S- and comma-shaped bodies (arrowheads), while 3’ signal of dp71 expression is found throughout the stroma and uretic buds. Dp140 expression persists in newborn WT kidney ( **C**), again restricted exclusively to distal/proximal convoluted tubules (insets **i** and **ii**). Dp71 is expressed more widely, found in collecting duct epithelia alongside rare focal regions of dp427 expression (inset **iii**). Images collected from multiplex-probed sections shown in ( Figure 4 and Supplementary figure 2. Scalebars: 100 µm. Subdivisions: 20 µm (panels **i**– **v**); 10 µm (insets). Full-size figure can be found in the *Underlying data* ^35^.

***Expression of dp140 in the developing kidney is focally restricted***. Dp140 is expressed within the developing (but not adult) kidney: Durbeej *et al.* ^18^ suggested this expression is moreover restricted to specific regions of the developing nephron. Our multiplex labelling confirmed this finding: as shown in Figure 12A, B, dp140 was found exclusively within the convoluted folds of comma and S-shaped bodies (dp71 conversely was distributed throughout the kidney stroma, with lower levels lining the ureteric buds). At this embryonic stage the nascent kidneys are markedly immature, and considerable (albeit degenerating) mesonephron remains. To investigate dp140 expression at a later stage, we used our triplex labelling ISH probes on neonatal kidney ( Figure 12C): again, this isoform was found only within specific regions of highly convoluted morphology. Given the absence of internal erythrocytes that would denote maturing glomeruli, these are most likely convoluted tubules, again supporting the findings of Durbeej and colleagues. Modest dp71 expression was present within collecting duct epithelia, but we also detected very low, discrete expression of dp427, something not reported previously ( Figure 12C iii). Contractile proteins are reported to be present within certain ductal structures of the kidney ^76^, and (as with eyelid fusion) such contractile apparatus may be accompanied by concomitant dp427 expression.

**Figure 13.**
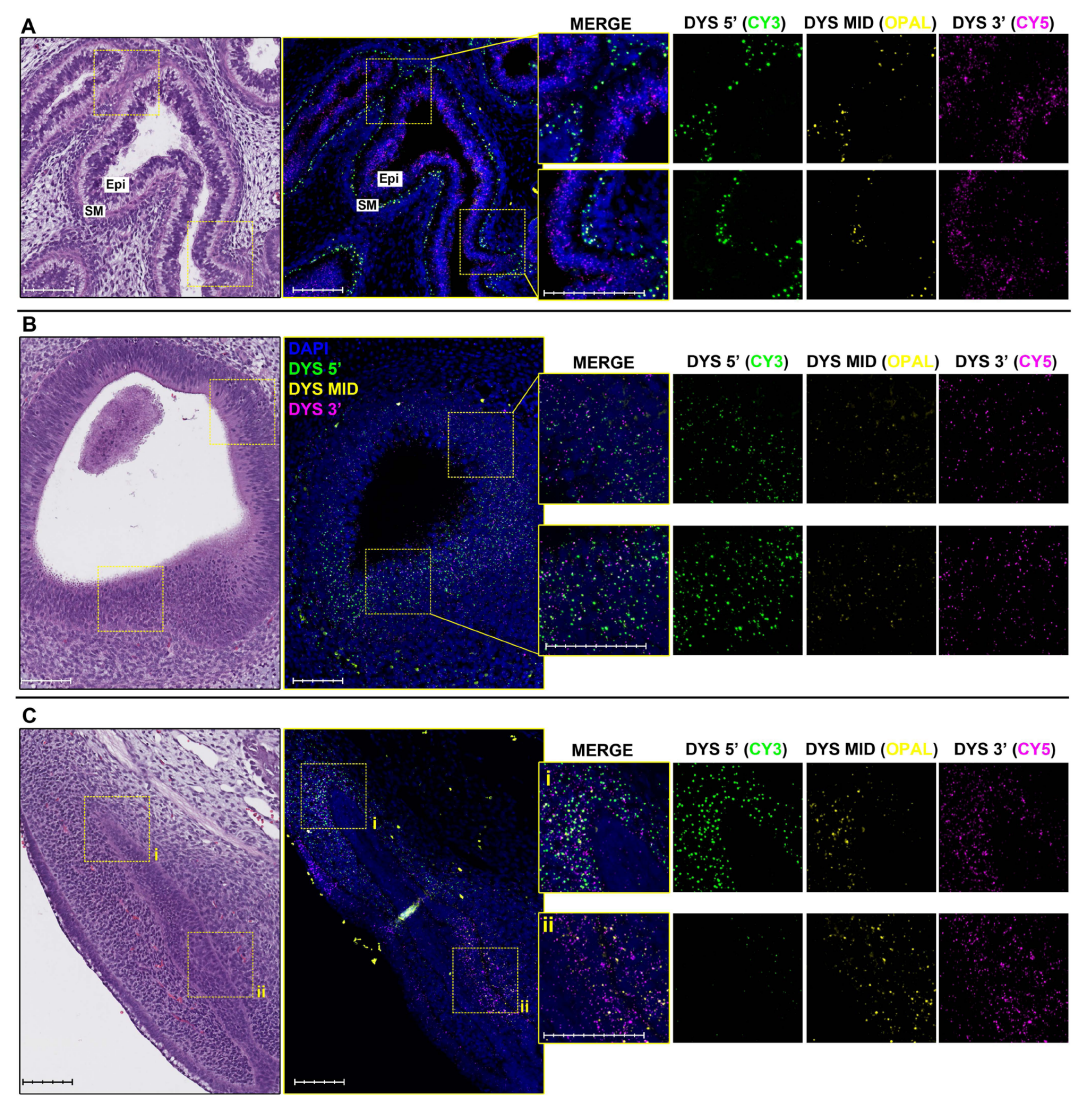
Dystrophin multiplex ISH in other tissues. Dystrophin expression in the embryonic lung ( **A**) is sharply partitioned: smooth muscle nuclei (SM) lining the nascent bronchioles express dp427, while the lung epithelium (Epi) expresses dp71 only. ( **B**) Dp427 is expressed in the migrating pseudostratified sensory neurons of the developing vomeronasal organ. ( **C**) Multiple dystrophin isoforms are expressed in the dental bud. Dp427 is robustly expressed in the condensing mesenchyme of the invaginated dental primordium wall ( **C**, inset **i**), while the nascent dental cap shows labelling indicating expression of dp140 (inset **ii**). Images collected from multiplex-probed sections shown in Figure 4 and Figure 5. Scalebars: 100 µm. Subdivisions: 20 µm (panels **i**– **v**); 10 µm (insets). Full-size figure can be found in the *Underlying data* ^35^.

***Other tissues express a range of dystrophin isoforms***. Dystrophin expression within other major organs chiefly reflects their smooth muscle and endothelial/epithelial content, demonstrated clearly by the lung ( Figure 13A). Nascent bronchioles were lined with dp427-rich cells, likely smooth muscle, while the surface layer of cells (developing lung epithelia) labelled strongly for dp71 as reported previously ^19^. Similar partitioning was found between the muscular wall of the developing stomach and the interior epithelial lining ( *Extended data*, Supplementary figure 5A ^60^), while the testis exhibited primarily peripheral dp71 consistent with encapsulating epithelium ( *Extended data*, Supplementary figure 5B ^60^). Despite strong cytoplasmic autofluorescence, no nuclear 5’ or middle probe staining was identified in the liver; however, 3’ labelling (consistent with dp71) was present ( *Extended data*, Supplementary figure 5C ^60^). Embryonic liver is host to several distinct cell types (including multiple blood cell lineages ^77^), and interestingly, dp71 labelling appeared restricted to specific nuclei. This isoform might therefore only be expressed in a distinct (minority) population of hepatic cells.

Some tissues present more diverse expression patterns, however: strong dp427 signal was found within the developing vomeronasal organ ( Figure 13B), potentially expressed by sensory neurons within the pseudostratified epithelium. More remarkably, both dp427 and dp140 are found in spatially distinct regions of the developing dental bud ( Figure 13C): here the full-length isoform was located in the condensing mesenchyme lining the bud wall, while dp140 expression was more focal, potentially demarking the nascent dental cap. While dp71 has been reported in dental primordia ^19, 40^, to our knowledge (similar to detection of dystrophin in developing bone, above), expression of these longer dystrophin isoforms in developing teeth is a novel finding.

## Discussion

Detection of dystrophin via ISH has historical precedent ^6, 18, 38, 39, 78^, and has greatly enhanced understanding of isoform expression in different tissues. Such approaches were limited by sensitivity (primarily resolving areas with many transcripts, such as nascent nuclear accumulations) and could moreover resolve only a single mRNA species at a time, precluding true comparisons between isoforms within a given tissue. We have previously pioneered a duplex single-transcript ISH approach to study dp427m in skeletal muscle ^42^, and demonstrated its efficacy: we believe the triplex work presented here represents another salient and novel application of this technology. The quantity of data collected from a modest selection of samples is substantial, and our unique multiplex approach also affords a very high signal-to-noise ratio (scattered non-specific staining is present at low levels, but the isoform-specific expression patterns observed are highly distinctive: co-localisation of two or three probes cannot easily be attributed to chance). We are able to detect even modest dystrophin expression within tissues otherwise impractical to study in isolation, such as embryonic bone. As we show, dystrophin is indeed expressed within this tissue, with different isoforms apparently present at distinct and specific locations within the developing limb (similarly, dystrophin is unexpectedly expressed during tooth development). We are further able to resolve spatially distinct distributions of dystrophin transcripts within sub-populations of cells, or even within cells themselves. We show that dp427 transcripts within embryonic primary muscle fibres appear to be targeted to MTJs (a feature absent in embryonic muscle of dystrophic animals), and within the eye, we show that retinal dp71 is enriched near the site of the nascent iris, and even reveal prominent dystrophin expression (potentially multiple isoforms) within tissues mediating eyelid fusion. Dystrophin is found in skeletal, cardiac and smooth muscle; brain, eye and peripheral nerve; kidney; lung; liver; blood vessels and multiple epithelial lineages; and, as we show here, also nascent bones and teeth. When examined at the whole-organism level it appears that relatively few tissues within the mammalian embryo do not express at least one dystrophin isoform.

A number of caveats must be acknowledged. Our multiplex approach uses inference by inclusion/exclusion criteria, and even triplex labelling does not permit distinction of all isoforms: presence of 5’ label unambiguously indicates expression of dp427, but does not distinguish between muscle, cortical or Purkinje isoforms: these differ only by the first exon (conferring 11, 3 and 7 unique N-terminal amino acids, respectively), while our 5’ probe spans exons 2-10. Middle probe in the absence of 5’ denotes dp260 or dp140, and 3’ label alone could indicate dp116, dp71 or dp40 (though tissue-specificity, low expression and qPCR corroboration partly address these issues). Our approach ostensibly assumes that multiple probes can bind to an appropriate transcript simultaneously: our data here and previously ^42^ strongly suggests this occurs, and labelling distributions within tissues of well-established expression (such as dp427m within skeletal muscle) are moreover highly consistent, but we cannot unambiguously discern dual- or triple-labelling of a single transcript from discrete labelling of individual isoforms, particularly within cell-dense tissues. High expression of dp140, for example, might mask modest co-expression of dp71, though abundant 3’ probe signal alongside only modest middle-probe signal (such as in the lens in Figure 8, or the nascent hypothalamus shown in Figure 9) is best interpreted as multiple isoforms. It is not at present known whether single cells can express multiple dystrophin isoforms simultaneously: both dp427 and dp71 are reported to be expressed in glia ^13, 71^, but not to single-cell resolution. While our data concerns mRNA only (which need not correlate directly with protein), multiple co-expressed isoforms would presumably compete for DAGC binding partners, rendering such expression detrimental under most contexts. Furthermore, the dystrophin gene lies on the X chromosome (single copy in males and subject to X-inactivation in females): only a single dystrophin locus is thus available for transcription within any given cell, and conflicting steric demands of initiation complexes and processive RNA polymerases would seem to preclude simultaneous expression. The first exon of dp71 lies a significant distance downstream of the initiation loci of dp427 (~2Mb) and dp140 (~1Mb), however, and this separation might permit a cell to commence transcription of a longer isoform several hours before subsequent cessation of dp71, potentially permitting a smooth transition between isoforms: single-cell RNAseq approaches will be required to answer such questions.

### Functional roles of dystrophin isoforms in development

Under the assumption that detected dystrophin transcripts are indeed translated to protein, our ISH data allows us to infer putative isoform-specific roles by considering shared features of the cell or tissue types in which certain isoforms are found. Dp427 is present in skeletal and smooth muscle as expected, but this isoform is also found focally expressed within the developing pons, thalamus and cerebellar primordium, within dorsal regions of the developing spinal cord, and within maturing chondrocytes of developing bone. Ostensibly an eclectic collection of tissue and cell types, this group nevertheless shares some key features: all are committed, post-mitotic lineages, and all are in the process of establishing long-term/permanent extracellular matrix interactions. Expression of dp71 is found within epithelial and endothelial cell populations, and within cell-dense regions such as the growth tips and the interzonal mesenchyme of the developing limbs, or the germinal layers of the mesencephalic roof and cerebellar primordium. Shared features here are proliferation and mobility: cells in the process of migrating or multiplying to line tissue surfaces, or to expand developing tissue territories (others have suggested similar roles for this isoform ^79, 80^). Finally, dp140 is highly expressed in the brain as expected (enriched in the diencephalon and within the germinal layer of the cerebellar primoridium, but also present within the developing telencephalon), and is found at specific locations in the kidney. This isoform is also present within the ventral spinal cord, at nascent skeletal entheses, and in the developing tooth: all regions of considerable cell- or tissue-level morphological plasticity.

Dystrophin is only one component of the DAGC (and not necessarily essential -in a subset of complexes, utrophin fulfils this role ^81^): many additional proteins can contribute to this complex, each of which might influence the behaviour of the resultant assembly ^24^. Some components (such as dystrobrevin ^82^) have multiple isoforms that are expressed in a tissue-specific manner, further complicating functional interpretations based on dystrophin alone. Nevertheless, interaction with dystroglycan and assembly of the DAGC requires only the dystrophin C-terminus, a domain present and (barring dp40) identical in all isoforms. Functional differences between dystrophin isoforms spetural perspective.

**Figure 14.**
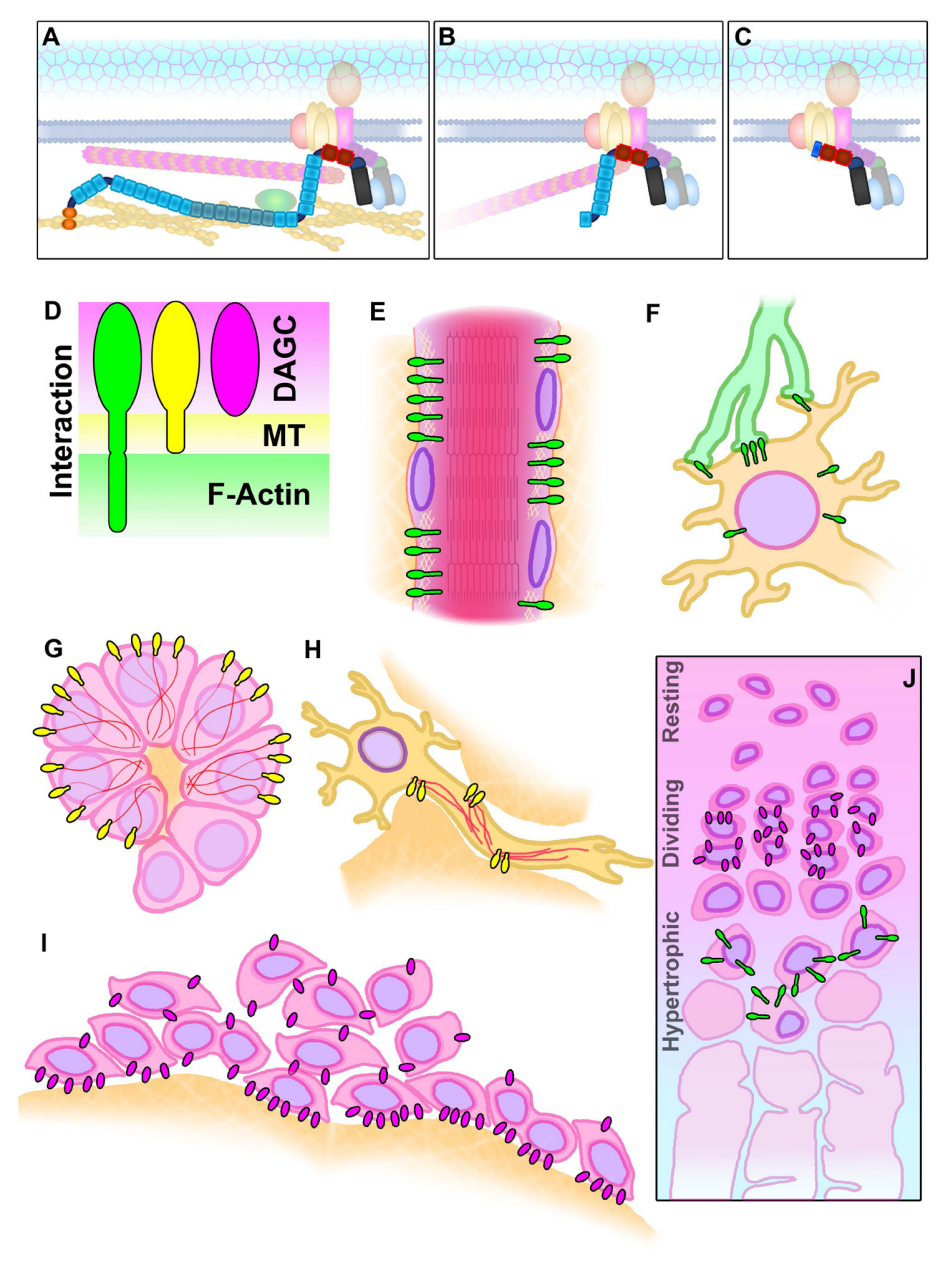
Isoform-specific functional roles. Full-length dp427 dystrophin ( **A**) binds cytoskeletal f-actin at two sites, interacts directly with microtubules and associates with dystroglycan to form the DAGC and establish a physical link with the ECM. Dp140 ( **B**) lacks both actin-binding domains but retains microtubule-binding and DAGC interactions. Dp71 ( **C**) can interact with DAGC components but cannot directly bind microtubules or cytoskeletal f-actin. The three isoforms can thus be grossly simplified by their structural interactions ( **D**). The highly stable physical interactions of dp427 mean this isoform is found in post-mitotic cells with permanent matrix interactions such as myofibres ( **E**) and neurons ( **F**). The more dynamic microtubule-based interactions of dp140 allow this isoform to influence localised cellular movement, mediating convoluted tubule curvature in the kidney ( **G**) and axonal migration in neural tissues ( **H**). Dp71 does not directly interact with any internal cytoskeletal components, thus is able to migrate freely within the membrane environment, allowing proliferation and migration and potentially aiding apical/basal determination ( **J**). Within developing bone ( **I**) dp71 allows chondrocytes within the proliferating zone to multiply, while expression of dp427 allows maturing chondrocytes to enter hypertrophy, define their niche and form lacunae. Full-size figure can be found in the *Underlying data* ^35^.

***Dp427***. Dp427 ( Figure 14A, D) is the only isoform with two discrete actin binding domains (at the N terminus and via spectrin repeats 11-17), and consequently this protein binds strongly to cytoskeletal f-actin, as well as microtubules (via repeats 20-23, a property shared with dp260 and dp140) and beta-dystroglycan (via the C terminus). As such, dp427 could be considered the most physically anchored isoform, by extension associated with establishing static cellular interactions (and potentially maintaining them under tension). Expression of dp427 might thus permit terminally differentiated, post-mitotic cells to refine their niche, anchoring themselves permanently and securely, and influencing the behaviour of their immediate extracellular environment. This is consistent with dp427 expression in muscle fibres ( Figure 14E), including the observed enrichment at presumptive MTJs, and similarly encompasses chondrocytes entering hypertrophy ( Figure 14J) and neurons ( Figure 14F). Notably, dp427 protein within neurons is found not at the migrating axon-derived pre-synaptic terminus but instead specifically at post-synaptic densities ^83, 84^ (where it both associates with GABA _A_ receptors in inhibitory synapses, and also contributes to excitatory post-synaptic transmission ^85^), again supporting a more static role (a similar argument might be made for dp427 expression at the neuromuscular junction in myofibres).

***Dp71***. Expression of dp71 seems primarily associated with more dynamic cell populations, where a high degree of cell motility or turnover is required. While this isoform still binds beta-dystroglycan and localises DAGC-associated proteins ^86^, domains capable of interaction with cytoskeletal components (f-actin and microtubules) are absent ( Figure 14C, D). Dp71 might therefore exhibit uniquely unrestricted mobility within the two-dimensional membrane-associated environment: properties perhaps well suited to more dynamic cellular behaviours like migration and proliferation, as found in the growth tips of developing bone ( Figure 14J). Indeed, unlike longer isoforms that might convey physical interactions across the plasma membrane, dp71 could be considered wholly responsive: expression of other isoforms might permit cells to influence their extracellular environment, but dp71 instead might only allow the extracellular environment to influence cells. Others have suggested an association of dp71 with terminally-differentiated lineages ^19^, which is not incompatible with our hypotheses: commitment to a specific cell fate does not imply withdrawal from mitotic division, and expression of dp71 might allow greater coordination of post-commitment proliferation and movement. This proposition matches our data, and readily explains the expression of this short isoform in migrating myoblasts ^21, 61^, where it would allow cells to be guided to appropriate locations by established matrix proteins, unhindered by internal cytoskeletal interactions. Similarly, the number of laminin-binding sugar epitopes carried by alpha-dystroglycan is modest in myoblasts, increasing only after commitment to the myogenic program ^87^, suggesting again that migrating myoblasts experience more permissive interactions with extracellular matrix components than do mature myofibres. A DAGC free of intracellular restrictions would also be expected to accumulate on whichever cell surface holds most extracellular interactions ( Figure 14I), potentially contributing to establishment of basal (high dp71) and apical (low dp71) faces, or aiding in clustering of tight junctions.

***Dp140***. Dp140, retaining microtubule binding in the absence of f-actin interactions ( Figure 14B, D), might consequently fall between these two extremes: either exerting mechanical influence across the membrane to the ECM in response to dynamic microtubule remodelling (unrestricted by internal stability of the actin cytoskeleton), or providing an ECM-anchored mooring to allow microtubule networks to exert mechanical influence within the cell. This isoform might thus play a key role in local morphological changes: smaller-scale cell/tissue remodelling or short-range movement. The presence of dp140 at attachment sites for ligaments, within cells mediating eyelid fusion, and within the convoluted comma- and S-shaped bodies of the kidney ( Figure 14G), supports this hypothesis. This functionality could readily extend to axonal migration as suggested by others ^17^, serving to provide ECM-dependent anchor points within the growing axon for microtubule-mediated growth-tip extension ( Figure 14H). Dp140 expression might thus indicate a nascent, presynaptic neuroblast stage, with a switch to dp427 only commencing once distal synaptic contact is achieved. As shown in Figure 11, the alar (dorsal) and basal (ventral) plates of the spinal cord exhibit robust but mutually exclusive expression of dp427 and dp140, respectively, and these differences mirror the functional specialisations of these regions. At this developmental stage the alar plate might be receiving early afferent connections from the periphery: dp427 would consequently be required for synapse formation. Conversely, developing neurons of the basal plate (conveying motor function) are extending axons from the spinal cord to innervate peripheral tissues: a migratory demand more suited to dp140. If this hypothesis is correct, one would expect earlier stages of spinal cord development (during axonal migration of spinal interneurons themselves) to exhibit greater and more widespread dp140 expression. This reasoning could be further extended to the brain, with regions undergoing active axonal migration (such as the developing hypothalamus and the germinal zone of the cerebellar primordium) being rich in dp140 but low in dp427, while deeper, more established (and presumably more mature) cell populations display the reverse. We note that dp71 appears to be expressed alongside dp140 in both developing metencephalon and diencephalon, suggesting that proliferation (or more extensive migratory processes) can occur simultaneously to any local cellular remodelling. The hypothalamus has been proposed to form via overlapping migratory and proliferative processes ^88^, and the germinal layer of the cerebellar primordium is host to actively proliferating Purkinje cells which then migrate outward to mature ^89^: both scenarios are compatible with our data. In neural lineages, therefore, isoform expression could represent a marker of maturity: a single Purkinje cell might express dp71 while proliferating, shift to dp140 for axonal migration and remodelling, before ultimately committing to an expression program of dp427 once established. Future studies, using embryos at different developmental stages, might help confirm this hypothesis.

### Isoform-specific transcriptional limitations

A further modulating constraint on isoform expression is provided by transcription time: the ~16 hours needed to produce a single full-length dystrophin transcript ^3^ is a physical limitation that cannot be circumvented, and is moreover comparable to typical mammalian cell doubling time ^90^. While recent studies suggest that for many genes, low levels of transcription continue even during mitosis ^91^, it is difficult to envisage how expression of dp427 might be retained throughout the interruptions of DNA replication and mitotic segregation. In essence, maintenance of cellular proliferation and concerted commitment to full-length dystrophin expression might be mutually exclusive: dp427 may be associated with terminal differentiation for the simple reason that only post-mitotic cells provide a sufficiently stable genomic environment for transcription (expression of dp260 or dp140, at ~10 and ~8 hours, respectively, might present similar challenges, while the ~1 hour needed for dp71 would not). Mitosis is also heavily-dependent upon orchestrated microtubule activity ^92^: a process potentially complicated by the presence of abundant membrane-localized microtubule recruiting domains found in dp427, dp260 and dp140 but not dp71. Dp71 is however thought to interact with microtubules via DAGC binding partners, and a role for this short isoform in mitosis itself has been proposed ^93^, suggesting the situation may be more nuanced (particularly given the number of reported dp71 splice variants ^80^). We further note that dp427 is transiently expressed in activated muscle satellite cells, apparently playing a role in asymmetric cell division ^94^. An association of dp427 with retained stem-ness is compatible with our proposed role for this isoform in niche-establishment, but transient expression is less readily explained. Satellite cells are quiescent rather than post-mitotic, however commitment to proliferation and subsequent differentiation occurs only after initial asymmetric division: transient expression of dp427 could thus mark the daughter cell returning to a quiescent, non-dividing state. The regenerative response to muscle damage is moreover not immediate: after activation, satellite cell proliferation typically commences >24 hours after injury ^94, 95^, sufficient time for transcription of modest numbers of dp427 mRNAs (indeed such lengthy transcriptional commitments could account for this lag in satellite cell response).

### Implications for dystrophin deficiencies

The expression patterns shown here reveal widespread and isoform-specific contributions of dystrophin during development, implying that the consequences of dystrophin deficiency might be broadly pleiotropic. DMD is primarily characterised by persistent myofibre damage and progressive muscle wasting, but patients also exhibit a range of neurodevelopmental and cognitive defects ^96^ as most mutations affecting sarcolemmal dp427m equally affect cortical dp427c (and the rare Purkinje isoform, dp427p). Similarly, mutations affecting dp140 typically include all dp427 isoforms and dp260, while mutations lying downstream of the first exon of dp71 will affect all dystrophin isoforms. Identifying unique isoform-specific contributions is thus challenging. Additive contributions clearly exist: mutations resulting in loss of dp140 as well as dp427 are not associated with more severe muscle damage, but they do exacerbate the cognitive phenotype ^97^. Notably (unlike muscle) these cognitive deficits do not appear to increase in severity with time, implying either passive, ongoing modulatory involvement of dystrophin, or a stage-sensitive developmental contribution. A role for dp427 in mature neurons, modulating GABA receptor clustering, fits the former hypothesis well, while a role for dp140 in mediating developmental axonal migration is compatible with the latter. It is clear dp140 is not essential for brain development, but its absence may compromise the organisation of developing axonal networks and might limit potential post-natal plasticity. Notably, dp140 persists at modest levels even in the adult brain, particularly within the cerebellum ^17^. Such site-specific expression implies a specific and persistent role, such as contributing to coordination and motor learning (though given the debilitating nature of DMD, it can be challenging to distinguish neural/central defects in muscle activity from muscle dysfunction itself). Dp140 is also expressed in the developing kidney and the eye, and as our data reveals, in embryonic teeth and bone (alongside dp427 and dp71). The absence of dp140 is not associated with any specific retinal, skeletal, dental or renal defects in DMD patients, suggesting that this isoform is not essential for their development. Some DMD patients exhibit abnormal electroretinograms ^98^, but loss of dp140 is typically accompanied by concomitant loss of dp427 and dp260. DMD boys also commonly display shorter statures than their peers ^99^, but formation and growth of bone is strongly influenced by reciprocal interaction with developing skeletal muscle ^64^, and this crosstalk likely weakens under dystrophic conditions. Indeed, the pleiotropic consequences of severe muscle disease likely makes it challenging to identify more subtle effects of dystrophin deficiency in other tissues: loss of ambulation itself increases risk of genitourinary conditions ^100^, and corticosteroids commonly given to DMD patients have many recognised side-effects, including stunted growth (further to that noted above) and osteoporosis. Animal models specifically deficient in dp140 alone (via mutation in the promoter or unique first exon) would help address these possibilities, though no such model currently exists.

Expression of dp71 revealed here is both widespread and tissue specific, associated strongly with developing epithelial/endothelial lineages and other zones with similar demands for proliferation and migration (growth tips and cartilaginous interzonal regions of the skeletal system, germinal layers of the brain, the growing lens and optic cup, and even within the liver). Such ubiquity of expression implies dp71 deficiency might be broadly debilitating. DMD boys with mutations affecting dp71 (in addition to dp427 and dp140) show more profound cognitive defects than those with mutations further towards the 5’ end of the gene ^101^. Dp71 is expressed in glia ^71^, and our data suggests a possible role for this isoform in proliferation/migration of multiple cell populations within the brain, thus absence of this short isoform could lead to both developmental defects (as proposed for dp140) and abnormal post-natal modulation (along with dp427). Similarly, short stature is commonly reported in DMD patients lacking dp71 ^102^, supporting a role for this isoform in migration and proliferation of bone mesenchymal cells. As with dp140 deficiency, it is challenging to dissect dp71-specific contributions away from other isoforms: only rare mutations to the promoter or unique first exon will affect this isoform in isolation, and no human cases have been documented (similarly, animal models lacking dp71 such as the *mdx ^3cv^* mouse typically also lack all longer isoforms). A specific dp71 null mouse model has however been generated ^19^: small stature is not reported, but this model does exhibit a brain phenotype, including cognitive defects (particularly spatial learning and inhibitory avoidance ^103^), and defects in CNS control of osmoregulation ^104^. Interestingly, the most markedly affected tissue is the eye, with retinal defects ^66, 105^ but also vascular defects ^106^ and cataracts ^68^. Dp71 is expressed in retinal Müller glia and vascular endothelia, but as shown in Figure 8, this isoform is also prominently expressed both within the crystalline lens fibres themselves, and within the epithelial cells that will ultimately surround the lens: disordered assembly of either cell population could well result in susceptibility to cataracts. DMD patients lacking dp71 might therefore be at greater risk of ocular disease, though long-term glucocorticoid treatment also increases cataract risk markedly ^107^, possibly masking mutation-specific contributions.

## Conclusions

This work represents more experimental proof of principle than detailed treatise: we have examined only a few individuals, and all were collected at the same developmental stage. Our findings are thus only a snapshot of developmental dystrophin expression, but nevertheless a snapshot of simultaneous expression of multiple, distinct and distinguishable isoforms at single-transcript resolution, something previously unachievable. Our ISH studies reveal novel sites of dystrophin expression (sites that merit wider study for dystrophic changes), and moreover suggest separable functional roles for each isoform. Our method will allow future studies to follow expression of dystrophin isoforms throughout the entire course of embryonic/foetal development, further refining our understanding of this remarkable gene.

## Data availability

### Underlying data

All data used in in the manuscript is available at the figshare repository within the project “Single transcript multiplex *in situ* hybridisation reveals unique patterns of dystrophin isoform expression in the developing mammalian embryo”.

Figshare: Dystrophin multiplex ISH: Raw image data. https://doi.org/10.6084/m9.figshare.11959056 ^53^.

This project contains raw 20x images used to prepare figures in this manuscript, and to generate tiled merges of the brain regions shown in Figure 9 and Figure 10.

Figshare: Canine skeletal muscle RNAscope raw data and analysis. https://doi.org/10.6084/m9.figshare.12015009 ^50^.

This project contains canine skeletal muscle raw image data, ImageJ macros (in .ijm format) and analysis.

Figshare: Dystrophin multiplex ISH: qPCR data. https://doi.org/10.6084/m9.figshare.12015021 ^59^.

This project contains raw qPCR data (skeletal muscle and embryonic dystrophin isoform expression, embryonic sex determination.

Figshare: Sanger sequencing data of embryos. https://doi.org/10.6084/m9.figshare.12015012 ^56^.

This project contains Sanger sequencing trace files used to determine embryo genotype.

Figshare: Dystrophin multiplex ISH: additional images. https://doi.org/10.6084/m9.figshare.12026535 ^54^.

This project contains additional 20x images collected from the embryo shown in *Extended data*, Supplementary figure 2 ^60^.

Figshare: Dystrophin single transcript multiplex in mammalian embryo: all manuscript figures (full size). https://doi.org/10.6084/m9.figshare.12124152 ^35^

This project contains all the figures presented in this manuscript at full-size 600dpi resolution.

### Extended data

Figshare: Dystrophin multiplex ISH: Extended data. https://doi.org/10.6084/m9.figshare.12040746.v1 ^60^.

This project contains the following supplementary figures:

- **Supplementary figure 1: positive and negative controls.** Positive control probes to (mouse) Polr2a, Ppib and UBC label canine muscle (A) while negative control probes (bacterial DapB) do not (B). Positive control probes also label canine embryonic brain (C) and eye (D).
- **Supplementary figure 2: Dystrophin multiplex ISH in an additional deltaE50-MD canine embryo.** Serial sections collected from a second deltaE50-MD canine embryo (day 31 of 63-day gestation) stained with haematoxylin and eosin (A) and with dystrophin multiplex ISH probes (B) as indicated (5’ probe: Cy3, green; middle probe: opal 520, yellow; 3’ probe: Cy5, magenta. Nuclei (DAPI): blue). ISH image shown is a composite of ~50 images collected at 5x objective. Regions subsequently examined in greater detail are indicated (cerebellar primordium, heart, kidney: yellow boxes). (C) generalised contrast-enhanced distribution map of probe-specific expression: 5’ probe signal is strongest in nascent musculature, heart, lung and cerebellar primordium, middle probe signal is found in ventricular brain regions and the kidney, while 3’ probe signal overlaps with 5’/middle probe, but is also found enriched at joint margins, dorsal and cranial ganglia and blood vessel walls. (D) Approximate plane of section for reference. Note strong autofluorescence from liver and blood vessels, particularly in middle probe (opal 520) signal. Scale bars: 5 mm (subdivisions 1 mm)
- **Supplementary figure 3: Dystrophin multiplex ISH in deltaE50-MD heart and skeletal muscle.** Embryonic cardiac muscle (A) and primary dorsal spinal muscle fibres (B) from deltaE50-MD embryos. Haematoxylin and eosin stained serial sections are shown (left panels) alongside equivalent regions probed for ISH. Regions of interest taken for enlarged channel-specific insets (rightmost panels) are indicated. Cardiac muscle exhibits labelling patterns characteristic of dp427 expression, though levels are modest. DeltaE50-MD spinal muscle expressed dp427 more robustly but sarcoplasmic foci indicating mature transcripts are absent, and no transcript accumulation at myotendinous junctions is observed. Images collected from multiplex-probed section shown in Supplementary figure 2. Scalebars: 100 µm. Subdivisions: 20 µm (larger panels); 10 µm (insets)
- **Supplementary figure 4: Dystrophin multiplex ISH in deltaE50-MD cerebellar primordium.** Cerebellar primordium in leftward lateral section, showing a gradient of dystrophin isoforms: cells of the marginal layer (potentially maturing Purkinje neurons) express dp427 (upper and middle panels) while cells of the germinal layer express dp140 and dp71 and the periventricular region itself expresses dp71 alone (lower panels). Images collected from multiplex-probed section shown in Supplementary figure 2. Scalebars: 100 µm. Subdivisions: 20 µm (larger panels); 10 µm (insets)
- **Supplementary figure 5: Dystrophin multiplex ISH in stomach, testis and liver.** (A) Embryonic stomach wall (WT) shows expression of dp427 in the smooth muscle lining (upper panels) while the nascent stomach epithelium expresses dp71 only. (B) Scattered 3’ foci consistent with modest dp71 expression are found throughout the developing testis, with greater levels found in the epithelial margin. (C) Despite high autofluorescence, no nuclear staining consistent with dp427 or dp140 expression is found in the embryonic liver. 3’ foci of dp71 expression are readily observed, found within discrete but robustly expressing cell populations. Scalebars: 100 µm. Subdivisions: 20 µm (larger panels); 10 µm (insets)

Figshare: Dystrophin multiplex ISH: Supplementary file 1. https://doi.org/10.6084/m9.figshare.12040755.v1 ^52^.

This project contains Supplementary file 1: Full-size images used for Figure 4, Figure 5 and Supplementary figure 2. A .zip format file containing dystrophin 3plex ISH images (collected with 5x objective and merged using the pairwise-stitching algorithm of Preibisch *et al.* ^51^) and slide scan images (collected at 20x) of matching serial sections stained with haematoxylin and eosin. Image key is provided as an accompanying text file.

Data are available under the terms of the Creative Commons Attribution 4.0 International license (CC-BY 4.0).
