# Supplementary material for: Single-transcript multiplex in situ hybridisation reveals unique patterns of dystrophin isoform expression in the developing mammalian embryo
Source: Wellcome Open Res. 2020 Jul 20;5:76. Originally published 2020 Apr 23. [Version 2] doi: 10.12688/wellcomeopenres.15762.2 (PMC7372313; doi:10.12688/wellcomeopenres.15762.2)
Supplement: Peer review history

# Peer review: Reviewer response for version 2

**DOI:** 10.21956/wellcomeopenres.17705.r39624

**Published:** 2020-07-20

**Authors:** Helge Amthor

**Affiliations:** 1 Université Paris-Saclay, UVSQ, Inserm, END-ICAP, 78000, Versailles, France

Authors responded conveniently to all raised concerns and improved the manuscript to the reviewer's satisfactions. I would like to congratulate authors for this beautiful study.

Is the work clearly and accurately presented and does it cite the current literature?

Yes

If applicable, is the statistical analysis and its interpretation appropriate?

Yes

Are all the source data underlying the results available to ensure full reproducibility?

Yes

Is the study design appropriate and is the work technically sound?

Yes

Are the conclusions drawn adequately supported by the results?

Partly

Are sufficient details of methods and analysis provided to allow replication by others?

Yes

Reviewer Expertise:

fundamental and translational myology

I confirm that I have read this submission and believe that I have an appropriate level of expertise to confirm that it is of an acceptable scientific standard.

# Peer review: Reviewer response for version 1

**DOI:** 10.21956/wellcomeopenres.17287.r38513

**Published:** 2020-06-08

**Authors:** Helge Amthor

**Affiliations:** 1 Université Paris-Saclay, UVSQ, Inserm, END-ICAP, 78000, Versailles, France

Beautiful and very comprehensive study using multiplex in situ hybridization for histological analysis of Dmd transcription in mature canine muscle from healthy dogs and deltaE50 muscular dystrophy dogs, as well as during end-embryonic/early fetal development using probes against 5', middle and 3' region.

 My concerns are generally minor and can be addressed by correcting the manuscript, adding some reasoning and adapting the discussion, without any experiments or new data required.

 Following general remarks:

 Authors used mouse specific probes, which in dogs are incompletely sequence specific, thereby risking lower sensibility and higher non-specific signal amplification. Non-specific signals could explain the surprising expression pattern in non-muscle cells, e.g. in connective tissue and cartilage. Why did authors perform the study on dogs and not mice? Why not designing dog specific probes? 

 Authors rely mainly on exploitation of one sagittal section from wildtype dog and one from dystrophic dog, which are not in the same plane, which makes comparison difficult. 

 Following specific remarks:

 Introduction - chapter Dystrophin multiplex ISH:

- Please give literature for note: "dp427 mRNAs (20-40 per myonucleus)"

- Authors state "Dystrophic muscle also reveals rare single cells labelled with 3' probe only....". The corresponding Figure 1G consists only of DAPI labelled nuclei and ISH signal. Single cells cannot be differentiated, and the term is confusing. I noted that the term "single cell" or "individual cells" was several used throughout the text in this sense, which is confusing and should be avoided.

MM

 Very detailed and complete.

 Result section

- Concerning qPCR results, authors state in the text: “In dystrophin samples, this 5’ to 3’ reduction was more prominent, again likely reflecting NMD-mediated degradation.” I cannot see this much difference between WT and dystrophic samples, if there is any.
- Figure 4: Comparison with H&E staining suggests very intense C3 and Opal520, which seems to me being red blood cells (reddish color in H&E), thus being autofluorescence.
- Figure legend of Figure 4 describes that 3’ probe is present in vessel walls. Because of low zoom, this is not/difficult to spot on the image.
- In figure 6, serial sections were used for H&E and IHS staining. Authors arranged images to imply the same field of view. This cannot be assured as different section level and absence of defined histological hallmarks. 
- There is no histological proof for the presence of myotendinous junctions in the images as absence of specific markers. Alternative explications for the local enrichment of ISH signal are possible. Authors should rather be careful to conclude about the presence of transcripts in MTJ. Maybe they could state/discuss/conclude, that focal transcript cumulation could be suggestive for higher expression in MTJ, however, more in-depth histological analysis will be required to ascertain a compartmentalized Dmd transcription along muscle fibers. 
- In figure legend 6, authors write: “Small foci of all three probes are found in the sarcoplasm”. The image does not allow to conclude of where exactly is the sarcoplasm. Of note, images A and C show that developing muscle fibers are rather sparse and surrounded by mesenchymal cells and cells that seem to be fat cells. This makes it even more difficult to conclude of whether transcripts are in muscle cells or adjacent in non-muscle cells. Authors should precise in the legend that (D) is tongue from healthy dog. 
- Authors refer to Supplementary figure 3A and 3B. I believe that this may be a typing error, as no such supplementary figures, and authors possibly refer to Supplementary figure 2A and 2B. 

Discussion

 I find the discussion lengthy and difficult to follow. It is an almost separate review. Authors should try to condense the discussion. Authors review in detail protein isoform expression. However, protein expression was not studied in the manuscript, and it remains to be determined whether ISH pattern corresponds with immunohistochemistry. I encourage authors to critically discuss results in view of MM.

Is the work clearly and accurately presented and does it cite the current literature?

Yes

If applicable, is the statistical analysis and its interpretation appropriate?

Yes

Are all the source data underlying the results available to ensure full reproducibility?

Yes

Is the study design appropriate and is the work technically sound?

Yes

Are the conclusions drawn adequately supported by the results?

Partly

Are sufficient details of methods and analysis provided to allow replication by others?

Yes

Reviewer Expertise:

fundamental and translational myology

I confirm that I have read this submission and believe that I have an appropriate level of expertise to confirm that it is of an acceptable scientific standard, however I have significant reservations, as outlined above.

# Response

**Published:** 2020-07-02

**Authors:** John Hildyard

Thank you for the kind words and insightful suggestions: as discussed below, we have implemented changes as suggested and agree that they strengthen the manuscript.

*Authors used mouse specific probes, which in dogs are incompletely sequence specific, thereby risking lower sensibility and higher non-specific signal amplification. Non-specific signals could explain the surprising expression pattern in non-muscle cells, e.g. in connective tissue and cartilage. Why did authors perform the study on dogs and not mice? Why not designing dog specific probes?*

 As we describe, the multiplex approach we use is a novel modification of RNAscope ISH (which typically does not permit multiple probes per transcript). Our initial investigations used probes directed to the 5’ and 3’ sequence only, and investigated expression of dp427m in mouse skeletal muscle (this work is cited within the manuscript, and images from these studies are shown in figure 1). We demonstrated that the long dp427 transcript readily permits multiple probes to bind, giving us the impetus for the broader and more nuanced work shown in this manuscript.

 The probes used for this initial work (one ‘off the shelf’, one custom) were designed to mouse sequences –indeed the RNAscope probe catalogue is predominantly mouse and human, with very few canine sequences- before committing to the expense of designing three custom canine probes, we investigated whether our mouse probes could be used in dog muscle. As shown in figure 2, ISH of canine muscle is comparable with ISH of mouse muscle (both healthy and dystrophic), and can be corroborated by qPCR. The studies in these dog embryos were facilitated therefore by the fact that we had already optimised the technique in mouse tissue, and confirmed comparable, robust and sensitive staining in this other species.

 The hybridisation strategy used by RNAscope ISH differs from conventional FISH: the method uses “ZZ pairs”: paired oligonucleotide probes that each recognise ~20-25 bases of sequence, and that must bind adjacently to form a amplification platform (see figure 1). Using 20 such pairs in sequence allows the approach to target ~1000 base sequences. Crucially, resolution of a discrete, single transcript ‘focus’ does not require all 20 pairs to bind, but binding of *any* pair requires very high sequence identity. Consequently, the approach appears to work extremely well with highly-conserved orthologues where significant stretches of identical sequence are present (as is the case with dystrophin in the regions targeted), while non-specific binding remains low (please note that all the mouse-specific positive control probes also gave the expected labelling pattern in canine tissue, while the negative control probes –to bacterial sequence- gave no non-specific labelling).

 Your question regarding mouse embryos is pertinent: indeed, we are currently finalising comprehensive studies in mouse embryos of varying developmental age (which will form the basis of a more extensive treatise). We observe the same staining patterns in the same locations –hence we judge the possibility of non-specific labelling in dog embryos to be very low.

 This manuscript is (as we note) essentially proof of principle: the canine embryos used were obtained serendipitously (via routine spaying of a pregnant carrier female for unrelated welfare reasons) and are very rarely available, but the larger size of these embryos allows anatomical detail to be resolved with greater clarity, and increases spatial separation between discrete tissue regions with differing expression patterns (such as those within the brain). However, the large size of the embryos has the disadvantage of costing substantially more (for appropriate probe incubation volumes) and creates massive file sizes for analysis which can be prohibitive; in short, there are advantages and disadvantages of using the dog embryos. The former is exploited in this current manuscript and the latter is being addressed with separate (and more extensive) work in the much smaller mouse embryos.

*Authors rely mainly on exploitation of one sagittal section from wildtype dog and one from dystrophic dog, which are not in the same plane, which makes comparison difficult.*

 Technically, three sections: one dystrophic and one wild type in the main manuscript, with a further dystrophic embryo in the extended data (see supplementary figure 2).

 As we note in our response to Professor Wilton, our sample pool was limited, and it was not possible to ensure identical orientation for all samples (though we endeavoured to use sections from similar regions). Ultimately, comparison of whole-embryo expression was not our primary goal: as noted within the manuscript, we do not expect to see gross differences in expression between healthy and dystrophic embryos. The deltaE50-MD mutation introduces premature termination codons (PTCs) in dp427 and dp260, but dp140 and dp71 will be unaffected. Moreover, while dp427/dp260 transcripts will be degraded by NMD, this occurs only after transcription is complete, allowing sites of expression to be readily identified by their strong nuclear signal.

*Introduction - chapter Dystrophin multiplex ISH:*

*Please give literature for note: "dp427 mRNAs (20-40 per myonucleus)"*

 This is the same study as that described in the previous sentence (our mouse RNAscope investigations with 5’ and 3’ probes). We have added the reference to this statement to make this clear.

*Authors state "Dystrophic muscle also reveals rare single cells labelled with 3' probe only....". The corresponding Figure 1G consists only of DAPI labelled nuclei and ISH signal. Single cells cannot be differentiated, and the term is confusing. I noted that the term "single cell" or "individual cells" was several used throughout the text in this sense, which is confusing and should be avoided.*

 An excellent point. By convention, nuclei can be considered to represent individual cells in most tissue, but the presence of multinucleate myofibres in skeletal muscle does complicate matters. Our experience of muscle histology (both healthy and dystrophic) leads us to conclude that these nuclei (typically found in disrupted regions) are more consistent with single cells lying outside of myofibres (and the reported presence of dp71 within myoblasts but not myonuclei is also consistent with this interpretation), but we accept that our descriptions lend unwarranted confidence to this conclusion. We have edited the manuscript accordingly, referring to these as nuclei rather than cells where appropriate.

*Result section*

*Concerning qPCR results, authors state in the text: “In dystrophin samples, this 5’ to 3’ reduction was more prominent, again likely reflecting NMD-mediated degradation.” I cannot see this much difference between WT and dystrophic samples, if there is any.*

 Our wording is perhaps confusing. Essentially, 3’ sequence of dp427 (which primarily represents mature transcripts) is present at much lower levels than 5’ sequence (mature and also nascent transcripts). This is consistent with most dp427 RNA being in nascent form (as we and others have shown previously).

 In dystrophic muscle where mature transcripts are rapidly degraded via NMD, levels of 3’ sequence are concomitantly lower still, while levels of 5’ sequence remain comparable.

 We have edited the text to the following:

 “In dystrophic samples, **levels of 3’ sequence appeared yet lower**, again likely reflecting NMD-mediated degradation.”

*Figure 4: Comparison with H&E staining suggests very intense C3 and Opal520, which seems to me being red blood cells (reddish color in H&E), thus being autofluorescence.*

 Correct, these are indeed blood cells, which do indeed autofluoresce markedly. The figure legend states ‘Note strong autofluorescence from liver and blood vessels’: this is an error which we have now corrected to “liver and erythrocytes”.

 Thank you for spotting this!

*Figure legend of Figure 4 describes that 3’ probe is present in vessel walls. Because of low zoom, this is not/difficult to spot on the image.*

 We agree, but we find this difficult to address to our satisfaction: this primarily illustrates the necessary compromises of image preparation for publication. RNAscope ISH allows detection of mRNA down to single-molecule resolution, but can readily be used in large sections (the embryos shown here are ~5cm in length). This presents unparalleled opportunities for study of gene expression, but brings concomitant challenges in data presentation.

 We have included figshare links to both the full-size figure 4 (as noted in the figure legend), and the original (5x objective) image used to generate figure 4 (which, if printed, would be more than a metre high): both these show the 3’ probe labelling of blood vessels beautifully, but we accept this is more challenging to see clearly following downsizing for online/pdf publication. We have edited the text to “major blood vessel walls”, which remain comparatively obvious even when downscaled. The figshare links are thus available for those readers who want to see the original data.

*In figure 6, serial sections were used for H&E and IHS staining. Authors arranged images to imply the same field of view. This cannot be assured as different section level and absence of defined histological hallmarks.*

 We are pleased that the reviewer recognises the care we took to align serial sections. Both the ISH and H&E images are collected from the same (serial) sections shown in figures 4 and 5: we state this clearly within the manuscript. Sections were collected at 4um thickness, and adjacent sections were used for comparisons, so differences in section level (which are unavoidable) should be modest: ~8um at most.

 We state plainly that the H&E and ISH images used for figures 6-13 are serial sections, (i.e. not the exact same tissue), but they are collected from the same regions (it is comparatively trivial to overlay the H&E and ISH images, thus the entire embryo effectively serves as a single, comprehensive histological hallmark). We have edited the text (in the methods section) to make it clear that directly adjacent serial sections were used, and that whole section images were overlaid to identify equivalent regions.

*There is no histological proof for the presence of myotendinous junctions in the images as absence of specific markers. Alternative explications for the local enrichment of ISH signal are possible. Authors should rather be careful to conclude about the presence of transcripts in MTJ. Maybe they could state/discuss/conclude, that focal transcript cumulation could be suggestive for higher expression in MTJ, however, more in-depth histological analysis will be required to ascertain a compartmentalized Dmd transcription along muscle fibers.*

 Thank you – yes, we agree: we identify these as myotendinous junctions as they occur at the extreme termini of nascent myofibres - myofibres themselves can clearly be identified both histologically (H&E) and via their prominent dystrophin ISH foci. Moreover these putative MTJ regions are typically in serried alignment, apparently interdigitating with adjacent non-muscle tissue. Morphologically they certainly appear to be MTJs, however we accept that we cannot make this assignation with 100% confidence, so we have adjusted the text accordingly (“presumptive MTJs” etc).

 Transcript accumulation is the most parsimonious explanation for the signal we observe: nuclear signals within nascent myotubes (large 5’ and middle probe foci) appear of comparable intensity regardless of position, arguing against an MTJ-proximal transcriptional increase (indeed we suspect levels of nascent transcription found within myonuclei may represent a physiological maximum, with levels of mature dp427 chiefly controlled post-transcriptionally). As can be seen in figure 6A&B, the intense signals at presumptive MTJs lie outside the nuclei, and indeed the most proximal nuclei lack the intense nuclear labelling, and thus are unlikely to expressing dp427. We have edited the text accordingly to better illustrate our reasoning here.

 “Notably, high numbers of these mature transcripts were found 20-40μm distant from dp427-expressing nuclei, concentrated at locations consistent with developing myotendinous junctions”

*In figure legend 6, authors write: “Small foci of all three probes are found in the sarcoplasm”. The image does not allow to conclude of where exactly is the sarcoplasm. Of note, images A and C show that developing muscle fibers are rather sparse and surrounded by mesenchymal cells and cells that seem to be fat cells. This makes it even more difficult to conclude of whether transcripts are in muscle cells or adjacent in non-muscle cells.*

 Thanks for this comment. We are confident that these signals lie within the sarcoplasm: typically modest fluorescence background in the opal520 channel (here assigned to middle probe) allows structures such as myotubes to be faintly discerned (we draw the reviewer’s attention to figure 6C where these structures are most visible). The presence of surrounding fat/mesenchyme serves only to reinforce this, as 5’ and 3’ probe foci are specifically not found within these regions (though some 3’ foci consistent with dp71 are observed).We accept we cannot make this claim with complete certainty, however, and have edited the legend text to the following:

 “Small foci of all three probes are found outside myonuclei, consistent with mature exported dp427 mRNAs within the sarcoplasm”

*Authors should precise in the legend that (D) is tongue from healthy dog.*

 Well spotted! Legend edited accordingly.

*Authors refer to Supplementary figure 3A and 3B. I believe that this may be a typing error, as no such supplementary figures, and authors possibly refer to Supplementary figure 2A and 2B.*

 With the greatest respect, the reviewer is in error here: supplementary figure 3 does indeed exist, as do supplementary figures 4 and 5. The approach taken by Wellcome Open Research is to rebrand supplementary data as ‘extended data’, all of which is uploaded separately to a repository (in this instance, figshare). The extended data section of the manuscript details the locations of the relevant files. Supplementary figure 3 presents 20x images taken from the appropriate regions of a second deltaE50-MD embryo (the embryo itself is, as the reviewer notes, depicted in supplementary figure 2).

 All of the figures shown within the manuscript (and indeed all of the underlying data and images used to generate these figures) are available at their original resolution from the same location. We would wholeheartedly encourage any interested readers to peruse these.

*Discussion*

*I find the discussion lengthy and difficult to follow. It is an almost separate review. Authors should try to condense the discussion. Authors review in detail protein isoform expression. However, protein expression was not studied in the manuscript, and it remains to be determined whether ISH pattern corresponds with immunohistochemistry. I encourage authors to critically discuss results in view of MM.*

 We apologise for the length of the discussion: it was certainly not our intention for this to be difficult to follow.

 We note that one section (the summary of dp427 functions) was in fact erroneously duplicated in the online version of the manuscript: this would certainly add to the length of the discussion (and make it more difficult to follow).

 We feel it is important to put our findings into context: while the reviewer correctly notes we investigate mRNA only (and we discuss the specific transcriptional limitations presented by such a large gene), the expression patterns revealed provide insights into possible functional roles for the final dystrophin protein isoforms.

 As described in our introduction, histological resolution of distinct dystrophin isoforms at the protein level is extremely challenging: C-terminal antibodies do not distinguish isoforms at all, while generation of N-terminal antibodies is hindered by the paucity of unique amino acids (indeed Professor Wilton notes that dp427m, c and p differ by only 3, 7 and 11 amino acids respectively, and dp140 has no unique amino acids whatsoever). Further challenges are the low abundance of non-muscle dystrophin protein, and the fact that most dystrophin antibodies do not work with fixed tissue.

 In lieu of a robust means to distinguish isoforms at the protein level, therefore, our work represents (we believe) the most comprehensive assessment of developmental dystrophin expression to date, identifying isoform-specific expression in several novel locations, and allowing possible functional roles for dystrophin proteins to be inferred. Discussion of these possible roles seems appropriate.

# Peer review: Reviewer response for version 1

**DOI:** 10.21956/wellcomeopenres.17287.r38516

**Published:** 2020-05-05

**Authors:** Steve D. Wilton

**Affiliations:** 1 Perron Institute for Neurological and Translational Science, Nedlands, WA, Australia; 2 Centre for Molecular Medicine and Innovative Therapeutics, Murdoch University, Perth, Australia

The work submitted by Hildyard and colleagues represents a massive undertaking to map expression (spatial and temporal) of the dystrophin gene isoforms at what is truly breathtaking resolution.

 The concept of 1 gene 1 protein in the 1960s was discarded with the appreciation of how many gene transcripts could be generated from one locus through alternative splicing. More recently the relevance of alternative splicing was questioned with reported of predominantly one major transcript from most genes and then you have the dystrophin gene. The largest in the human genome with multiple transcripts generated through the use of alternative promotors (alternative splicing plays a minor role).

 Hilyard and colleagues have developed single transcript isoform specific in situ hybridization to follow complex expression in a developing embryo. The major muscle specific full length dp427 isoform had received most attention due to dystrophin mutations that lead to Duchenne muscular dystrophy and this work follows expression of this isoform and all the others in multiple tissues in  developing canine (normal and dystrophic) embryos.

 The manuscript is very well written, clearly laid out and logical. The data is both breathtaking and compelling and will set the standard for future studies.

 I only have some very minor comments/suggestions: 

 First paragraph Intro. There is mention of dp427m, do427c and dp429p. It could be worth mentioning (after the three discrete promotors) that these massive proteins differ by 3,7 and 11 amino acids difference at amino terminus.

 In the figures, the yellow text (dp140) is hard to read (at least on my screen). Could this be made clearer?

 I am not sure if it is significant but could the authors comment on why the approximate plane of section was different between normal and dystrophic embryos (fig 4 and 5).

 Figure 6D. Please specify this was from a healthy animal (it does mention this in the text).

 In discussion on page 25 the authors mention the inability to distinguish muscle, cortical and Purkinge isoforms. Perhaps this would be an appropriate place to mention the 3, 7, 11 amino acid differences.

 A little later they mention “A significant distance lies between the dp427 and

 dp140 initiation loci and the first exon of dp71”. Could they please give a rough indication in hundreds of kilobases.

 All in all, probably one of the longest manuscripts I have ever reviewed but certainly one of the most informative and impressive.

Is the work clearly and accurately presented and does it cite the current literature?

Yes

If applicable, is the statistical analysis and its interpretation appropriate?

Yes

Are all the source data underlying the results available to ensure full reproducibility?

Yes

Is the study design appropriate and is the work technically sound?

Yes

Are the conclusions drawn adequately supported by the results?

Yes

Are sufficient details of methods and analysis provided to allow replication by others?

Yes

Reviewer Expertise:

Antisense manipulation of gene expression

I confirm that I have read this submission and believe that I have an appropriate level of expertise to confirm that it is of an acceptable scientific standard.

# Response

**Published:** 2020-07-02

**Authors:** John Hildyard

We thank the reviewer for his excellent summary and supportive assessment, and are delighted he finds this work as interesting as we do.

 The reviewer’s suggested changes are appreciated: we have endeavoured to address each point raised as follows:

*It could be worth mentioning (after the three discrete promotors) that these massive proteins differ by 3,7 and 11 amino acids difference at amino terminus.*

 We have added reference to the amino acid differences between dp427m, c and p to the introduction, on page 5 (study of dystrophin isoform expression).

*In the figures, the yellow text (dp140) is hard to read (at least on my screen). Could this be made clearer?*

 An excellent suggestion: figures 3, 8 and 11 have been edited to make the yellow ‘dp140’ text more legible.

*I am not sure if it is significant but could the authors comment on why the approximate plane of section was different between normal and dystrophic embryos (fig 4 and 5).*

 In honesty, more necessity than design. The canine embryos used for this study were obtained serendipitously (via routine spaying of a pregnant carrier female for unrelated welfare reasons): our sample numbers are necessarily modest as a consequence. Preparation and processing of comparatively large (~5cm long) and intricate specimens is moreover challenging, and it was not possible to ensure identical orientation in all cases. The embryo sections used were ultimately selected on criteria of well-preserved morphology and relevant tissue content, rather than identical plane of section. Moreover, as we show in our manuscript, both normal and dystrophic embryos can be used to determine sites of expression: dp140 and dp71 will not be affected by the deltaE50-MD mutation, while dp427/dp260 expression (which would be subject to nonsense mediated decay (NMD)) can be recognised by nuclear expression by virtue of the lengthy transcription time. In essence we are able to present two complementary planes of section, while still identifying specific dystrophic changes (such as loss of mature transcript accumulation at myotendinous junctions).

*Figure 6D. Please specify this was from a healthy animal (it does mention this in the text).*

 Thank you. We have added “(WT)” to the appropriate location in the figure legend.

*In discussion on page 25 the authors mention the inability to distinguish muscle, cortical and Purkinge isoforms. Perhaps this would be an appropriate place to mention the 3, 7, 11 amino acid differences.*

 An excellent suggestion. We have edited the text as follows:

 “but does not distinguish between muscle, cortical or Purkinje isoforms: **these differ only by the first exon (conferring 11, 3 and 7 unique N-terminal amino acids, respectively), while our 5’ probe spans exons 2-10.**”

*A little later they mention “A significant distance lies between the dp427 and*

*dp140 initiation loci and the first exon of dp71”. Could they please give a rough indication in hundreds of kilobases.*

 Thank you for this suggestion: text changed as follows.

**“The first exon of dp71 lies a significant distance downstream of the initiation loci of dp427 (~2Mb) and dp140 (~1Mb)**, however, and this separation might permit…”
